# Rational Design of Phosphorylation-Responsive Coiled Coil-Peptide Assemblies

**DOI:** 10.1021/acssynbio.3c00064

**Published:** 2023-03-29

**Authors:** Harry F. Thompson, Joseph L. Beesley, Hannah D. Langlands, Caitlin L. Edgell, Nigel J. Savery, Derek N. Woolfson

**Affiliations:** † School of Biochemistry, University of Bristol, University Walk, Bristol, BS8 1TD, U.K.; ‡ School of Chemistry, University of Bristol, Cantock’s Close, Bristol, BS8 1TS, U.K.; § Bristol BioDesign Institute, School of Chemistry, University of Bristol, Cantock’s Close, Bristol, BS8 1TS, U.K.

**Keywords:** coiled coil, inducible conformational switch, phosphorylation, protein design, rational peptide design, synthetic biology

## Abstract

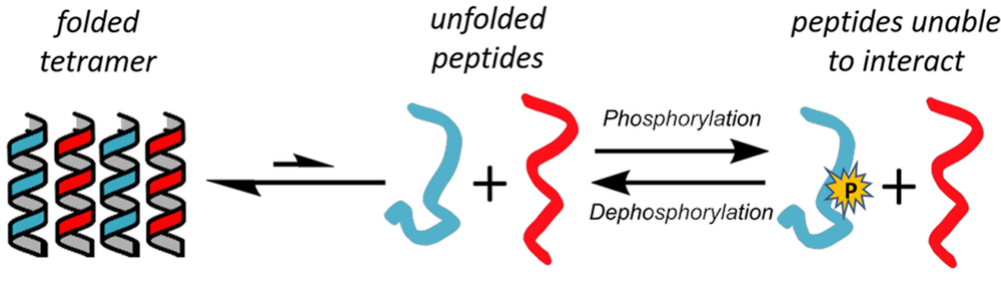

*De novo* peptides and proteins that switch state in response to chemical and physical cues would advance protein design and synthetic biology. Here we report two designed systems that disassemble and reassemble upon site-specific phosphorylation and dephosphorylation, respectively. As starting points, we use hyperthermostable *de novo* antiparallel and parallel coiled-coil heterotetramers, *i.e.*, A_2_B_2_ systems, to afford control in downstream applications. The switches are incorporated by adding protein kinase A phosphorylation sites, R-R-X-S, with the phosphoacceptor serine residues placed to maximize disruption of the coiled-coil interfaces. The unphosphorylated peptides assemble as designed and unfold reversibly when heated. Addition of kinase to the assembled states unfolds them with half-lives of ≤5 min. Phosphorylation is reversed by Lambda Protein Phosphatase resulting in tetramer reassembly. We envisage that the new *de novo* designed coiled-coil components, the switches, and a mechanistic model for them will be useful in synthetic biology, biomaterials, and biotechnology applications.

## Introduction

Protein–protein interactions (PPIs) are involved in a wide range of biological processes.^1^ Many PPIs are complex and multifactorial making them challenging to exploit predictably in synthetic biology. Increasingly, through *de novo* peptide and protein design, it is becoming possible to generate synthetic polypeptides that mimic and augment natural protein structures and functions, including PPIs.^2−4^ In principle, the relative simplicity of *de novo* peptides and proteins makes it easier to predict the outcomes of altering them than for natural proteins. Coiled coils (CCs) are one of the most prevalent and versatile PPI motifs.^5^ Moreover, they are amenable to rational and computational design.^4,6^ Here, we describe the design and characterization of two *de novo* designed CC systems that incorporate phosphorylation sites to allow for enzymatic control of their disassembly and reassembly.

CCs comprise two or more α helices that wrap around each other to form supercoiled quaternary structures.^5^ These structures are directed by sequence patterns of hydrophobic (***h***) and polar (***p***) residues, most commonly as heptad repeats, ***hpphppp*** (often denoted ***abcdefg***). This relative simplicity has allowed the elucidation of sequence-to-structure relationships for CCs, which have been exploited in *de novo* peptide and protein design.^6^ For example, hydrophobic residues at the ***a*** and ***d*** sites of the heptad repeat direct the folding and assembly of amphipathic α helices, and the sizes and configurations of the bundles formed can be defined by using different combinations of side chains at ***a***, ***d***, ***e***, and ***g***. In this way, designs have been achieved and characterized through to X-ray crystal structures for homomeric and heteromeric assemblies, all-parallel or antiparallel arrangements of helices, and oligomeric states spanning dimers to octamers. These provide toolkits of *de novo* CC modules.^4,6^ In turn, such toolkits have been used to guide the assembly of a wide variety of multiprotein complexes and supramolecular structures both *in vitro* and, increasingly, *in vivo*.^7−11^ This has included reconstituting split enzymes, engineering new transcription factors, and constructing protein origami, fibers, and cages for subcellular applications.^9−16^ However, the vast majority of these *de novo* CC modules are static, single-state assemblies. Thus, a challenge in CC design—and for protein design generally^4,17−21^—is to incorporate the dynamics and switching behaviors that are often key to the activities of natural proteins.^1^

A number of one-way CC-based switches have been made that respond to changes in heat, disulfide-bond reduction, and metal binding.^22−27^ In addition, photoswitchable CCs have been demonstrated,^28^ although these require non-natural amino acids that may hamper their use in living systems. A CC-based hairpin that opens in response to a target protein binding to its loop region has also been produced.^29^ Most recently, two pH-inducible CC switches have also been reported.^18,30^ An alternative and less-explored approach is to incorporate sites for post-translational modifications (PTMs) into CCs and to bring about reversible switching enzymatically. Phosphorylation is a widespread PTM with estimates that over half of all eukaryotic proteins are targeted.^31^ In turn, phosphorylation is frequently used to modulate natural PPIs.^32^ Minimal phosphorylation motifs have also been examined in various engineered and *de novo* peptide systems.^33−37^

Natural protein kinases modify various phosphoacceptor residues (predominantly Ser, Thr and Tyr in eukaryotes, plus His and Asp in prokaryotes) within specific recognition sites. For example, the eukaryotic cAMP-dependent protein kinase A (PKA) targets a short, well-defined substrate motif, Arg-Arg-Xaa-Ser/Thr, where Xaa is any amino acid. This simple motif has been incorporated directly into various CC and similar designs. For example, reversible stabilization and destabilization of CC heterodimers has been achieved through phosphorylation by PKA;^38^ a phosphorylation-dependent switch for membrane targeting has been designed using a *de novo* designed heterodimeric CC as the switchable PPI domains;^39^ and phosphoswitchable elements have been introduced into the *de novo* helical-bundle strand-displacement system, LOCKR.^19,40,41^

For the work reported here, we set out to design a reversible Boolean switch between completely folded and completely unfolded states of *de novo* CC heterotetramers brought about by phosphorylation. We targeted heterotypic systems because of the added control that they give over assembly.^10,42^ And we selected heterotetramers over heterodimers because of their potentially increased cooperativity of assembly, which we reasoned would give the more-sensitive responses required for designing systems with high dynamic ranges for future applications.^10^ For our design strategy, given the negative charge and bulk of the phosphoryl group, we placed the phosphoacceptor residue at central ***a*** or ***d*** sites of the *de novo* CCs to maximize the impact of the enzymatic modifications on CC assembly. This led to two new designs for a parallel and an antiparallel heterotetrameric CC, both of which accommodate phosphorylation sites in their folded states. Both designs respond rapidly to addition of kinase or phosphatase, producing two reversible on–off switches. Finally, based on experimental parameters, we develop a mechanistic model for one of the switches to aid future applications in synthetic and chemical biology.

## Results and Discussion

### Rational *de novo* Design of an Antiparallel A_2_B_2_ Heterotetramer

Our starting scaffolds for the design of phosphoswitchable peptides were parallel and antiparallel A_2_B_2_ heterotetramers. For the parallel scaffold, we already had a published variant of the well-characterized and hyperthermostable CC-Tet homotetramer, CC-Tet-A_2_B_2_, Table 1.^10,43^ However, when we began this work, no suitable *de novo* designed antiparallel CC heterotetramer was available.

**Table 1:**
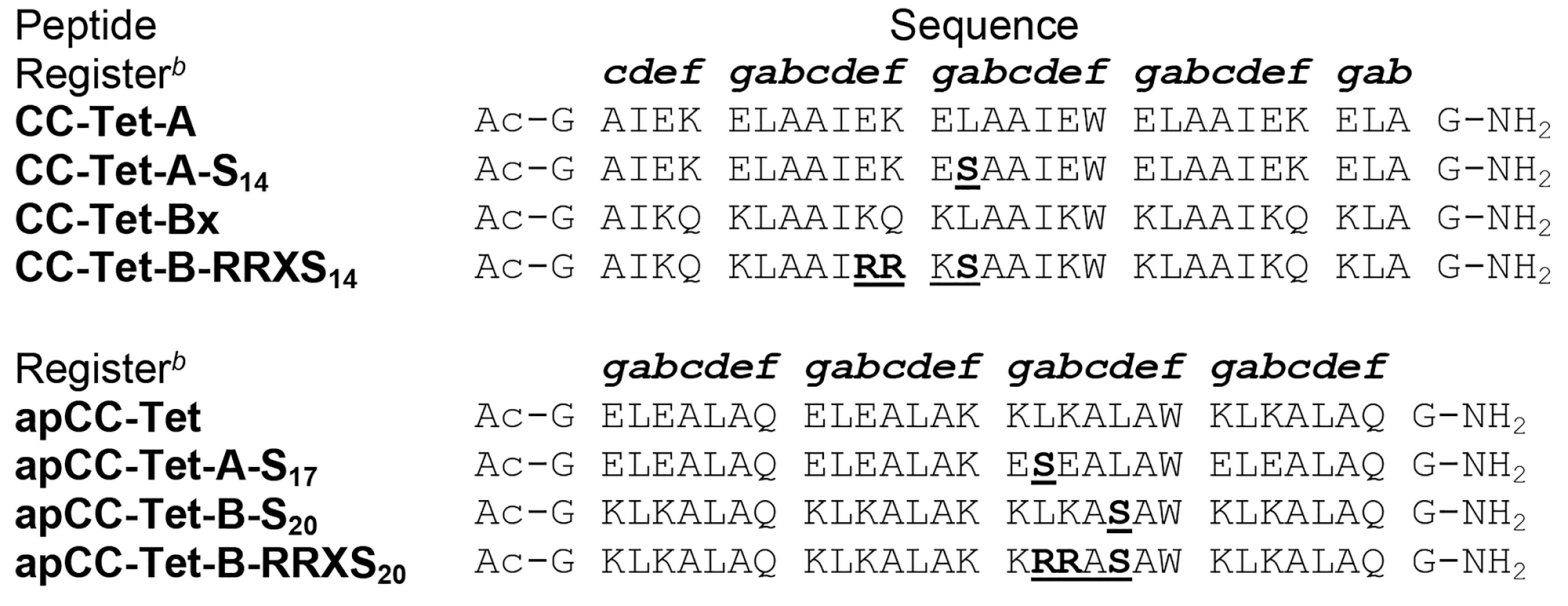
Sequences of Heterotetramer Peptides^a^

a All peptides were N-terminally acetylated and C-terminally amidated.

b Heptad registers are given above the sequences. Peptides are in **c**- or **g-**register, which refers to the heptad positions of the first residue of the peptide sequence excluding the capping Gly residues. Introduced sequence variations are highlighted in bold and underlined.

To address this gap, we set out to produce such a system, *i.e.*, apCC-Tet-A_2_B_2_, based on an antiparallel homotetramer, apCC-Tet.^44^ By modeling in the computational protein-design tool ISAMBARD,^45^ we generated a system analogous to CC-Tet-A_2_B_2_, comprising acidic (A) and basic (B) peptides and an otherwise unchanged hydrophobic core (Figure S1 and Table S1). We called these peptides apCC-Tet-A1 and apCC-Tet-B1 (Table S1). Both peptides were made by Fmoc solid-phase peptide synthesis, purified by HPLC, and confirmed by mass spectrometry (Figure S2).

When mixed, peptides apCC-Tet-A1 and apCC-Tet-B1 gave a hyperthermostable, α-helical complex (Figure S3). However, the individual peptides were also stably folded (Figures S3 and S4), which was incompatible with our goal of an on–off switch. Therefore, to destabilize the homomeric states of these designs, we introduced serine (Ser, S) residues at an ***a*** site of the apCC-Tet-A1 peptide to give apCC-Tet-A-S_17_, and the complementary ***d*** site of apCC-Tet-B1 to give apCC-Tet-B-S_20_ (Table 1). We chose Ser for two reasons: first, Ser is one of the more-tolerated polar residues at ***a*** and ***d*** sites of antiparallel coiled-coil tetramers;^46^ and second, it fitted our aim of introducing a natural phosphorylation site later in the design process.

**Figure 1:**
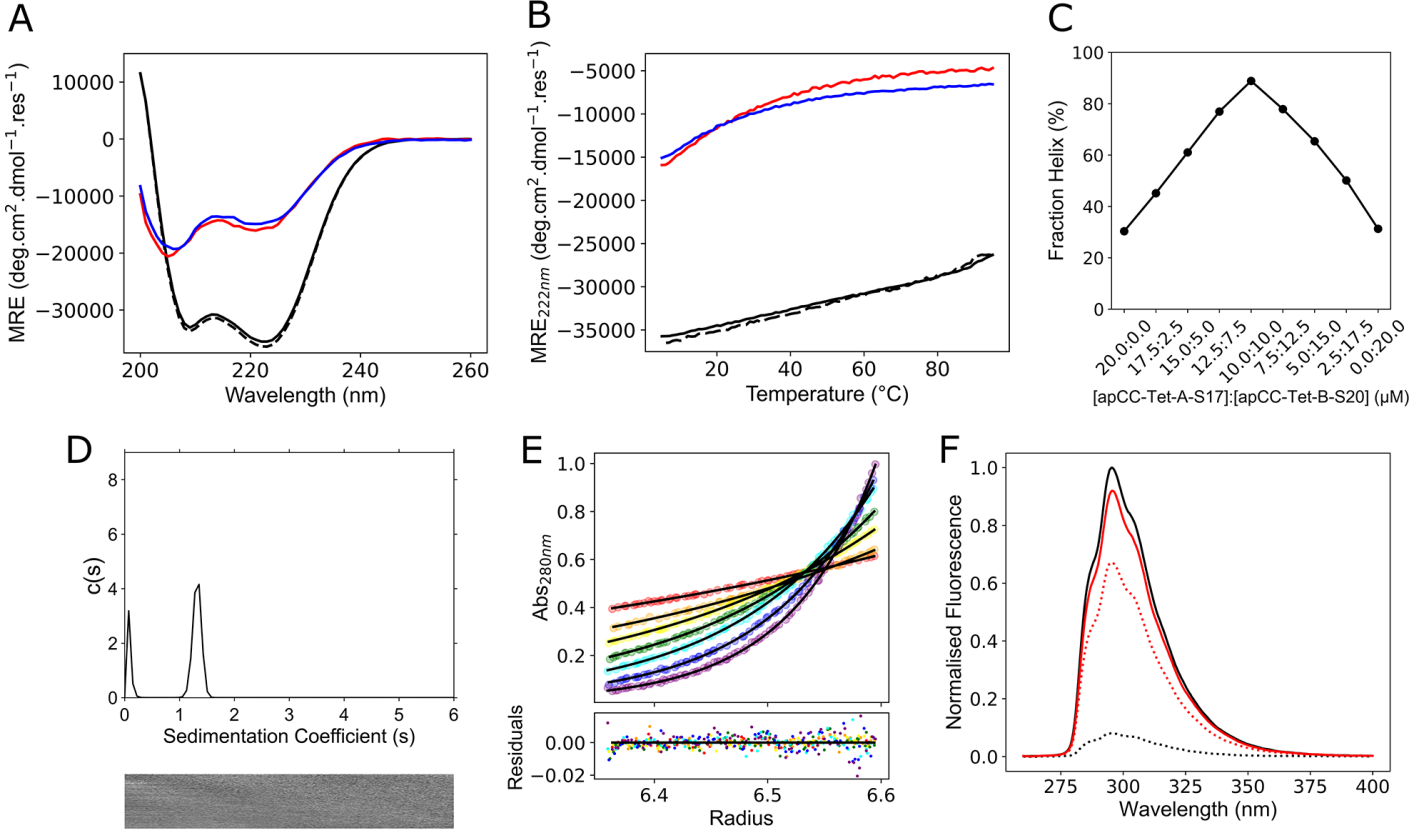
*De novo* designed peptides apCC-Tet-A-S_17_ and apCC-Tet-B-S_20_ form an antiparallel heterotetramer apCC-Tet-A_2_B_2_ in solution. (A) Representative CD spectra at 5 °C and (B) variable temperature CD measurements monitoring MRE_222_ (mean residue ellipticity at 222 nm) between 5 and 95 °C. Key: apCC-Tet-A-S_17_ (red), apCC-Tet-B-S_20_ (blue), mixture apCC-Tet-A-S_17_ plus apCC-Tet-B-S_20_ (black, with postmelt scan and refolding measurements dashed). (C) Job plot showing fraction helix values calculated for different ratios of apCC-Tet-A-S_17_ plus CC-Tet-B-S_20_, maintaining a total peptide concentration of 20 μM. (D) Sedimentation velocity (SV) and (E) sedimentation equilibrium (SE) analytical ultracentrifugation (AUC) data for the mixture of apCC-Tet-A-S_17_ plus apCC-Tet-B-S_20_. Analyses of these data returned molecular weights of 13,787 Da (4.3× monomer mass) and 12,589 Da (3.9× monomer mass, 95% confidence limits 12572–12608 Da), respectively. (F) Fluorescence-quenching assay for labeled apCC-Tet-B-S_20_ (with 4CF at position 2 or 25), with or without apCC-Tet-A-S_17_ (with SeMet at position 4). Key: apCC-Tet-B-S_20_-4CF_25_ (solid black line), apCC-Tet-B-S_20_-4CF_2_ (solid red line), apCC-Tet-B-S_20_-4CF_25_ plus apCC-Tet-A-S_17_-SeMet_4_ (dashed black line), apCC-Tet-B-S_20_-4CF_2_ plus apCC-Tet-A-S_17_-SeMet_4_ (dashed red line). Conditions: All measurements were performed in phosphate buffered saline (PBS) (pH 7.4), except (F), which was performed in phosphate buffer (pH 7.4). CD spectra and thermal denaturation curves were recorded at 10 μM of each peptide, SV at 70 μM of each peptide, SE at 35 μM of each peptide, and quenching experiments at 50 μM of each peptide.

For the individual Ser-containing peptides, circular dichroism (CD) spectra and thermal unfolding experiments confirmed that they remained mostly unfolded at 10 μM peptide concentration (Figures 1A,B, and S5). However, when mixed, the peptides formed an α-helical, hyperthermostable tetramer (Figures 1A,B,D,E). A Job plot constructed from CD data recorded at different ratios of the two peptides revealed that this complex had a 1:1 stoichiometry of the acidic and basic chains (Figures 1C and S6). To confirm the antiparallel orientation of apCC-Tet-A_2_B_2_ designs, we also made variants of apCC-Tet-A-S_17_ with seleno-methionine (SeMet) at position 4, and apCC-Tet-B-S_20_ with 4-cyano-l-phenylalanine (4CF) at positions 2 or 25 (Table S1). 4CF is fluorescent, and its fluorescence can be quenched by SeMet when the two side chains are brought in proximity upon coiled-coil assembly.^47^ Therefore, for the two 4CF variants of the B peptide, that modified at position 2 should be quenched by the modified A peptide if the assembly has all-parallel helices; and that with 4CF at position 25 should be quenched in an antiparallel assembly. We found the latter, confirming the antiparallel nature of the interaction (Figure 1F).

In summary, apCC-Tet-A-S_17_ and apCC-Tet-B-S_20_ combine in solution to form a stable, cooperatively folded antiparallel heterotetramer. On this basis, and for simplicity, we renamed these apCC-Tet-A and apCC-Tet-B, respectively, and the resulting complex apCC-Tet-A_2_B_2_. This is a new *de novo* designed CC component, although we have recently reported another example of this design target, which we have named apCC-Tet*-A_2_B_2_.^48^ These recently published designs differ from the original apCC-Tet systems in two ways: the heterotetramer, apCC-Tet*-A_2_B_2_, has 3 heptads, which limits design variations; and the residues used for core packing at the ***a*** and ***d*** sites are different, with the apCC-Tet designs having Leu-Leu cores and the apCC-Tet* designs having Leu-Ile cores. Among other things, we anticipate that these will be useful for mimicking or substituting for antiparallel PPIs, which are common in natural systems.^46^

### Engineering CC Heterotetramers Harboring PKA Sites

With the antiparallel heterotetramer, apCC-Tet-A_2_B_2_, and the previously designed parallel heterotetramer, CC-Tet-A_2_B_2_,^10^ in hand, we moved onto the design of phosphoswitchable assemblies for both orientations. In the absence of high-resolution structures for either heterotetramer, we investigated the possible impacts of adding the PKA consensus motif, Arg-Arg-Xaa-Ser (R-R-X-S), to these assemblies using *in silico* parametric modeling, which is advanced and reliable for CCs.^45,49,50^

#### Modeling

**Figure 2:**
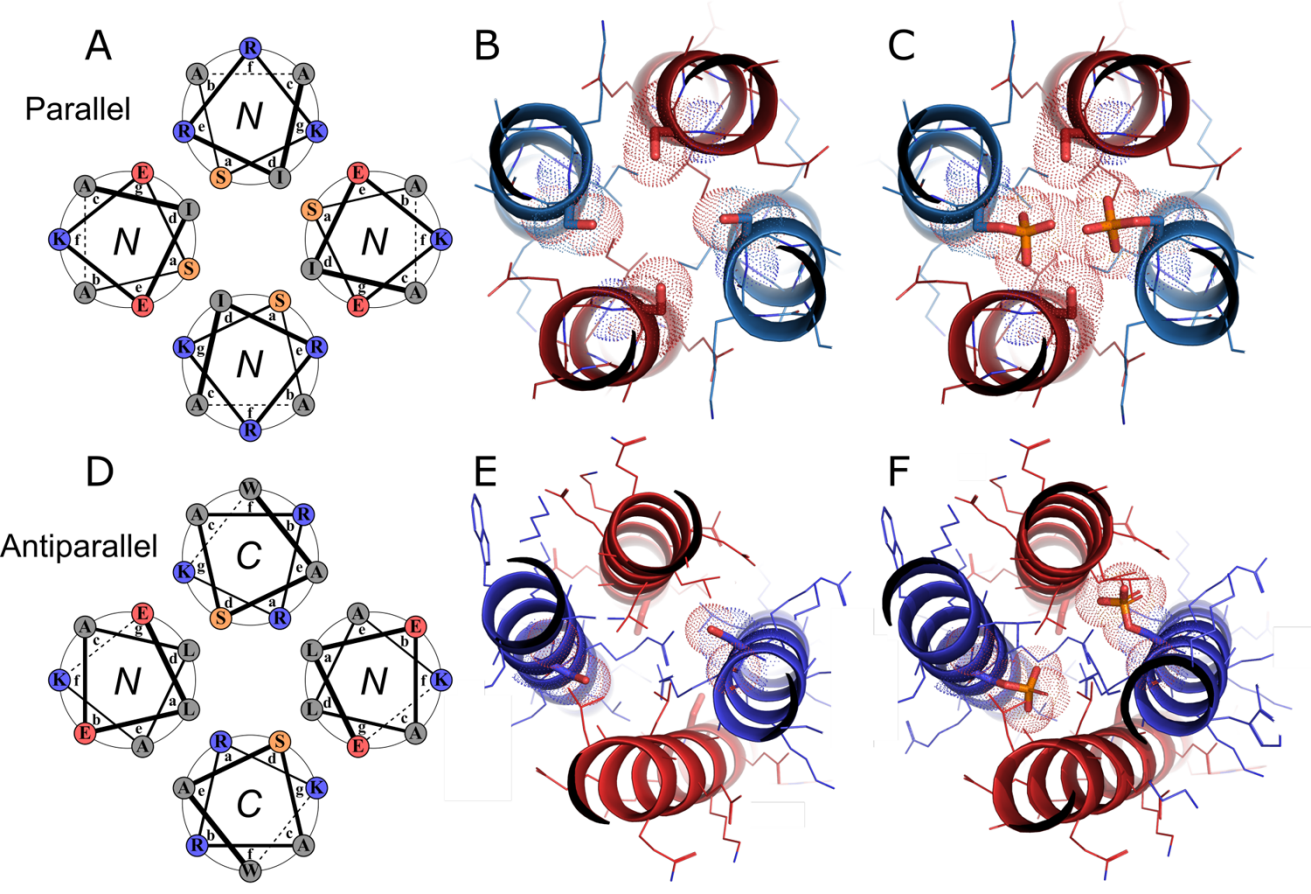
Design and modeling of serine- and phosphoserine-containing A_2_B_2_ heterotetramers. (A–C) The parallel CC-Tet-A-S_14_ (red) plus CC-Tet-B-RRXS_14_ (blue) combination. (D–F) The antiparallel apCC-Tet-A-S_17_ (red) plus apCC-Tet-B-RRXS_20_ (blue) combination. (A,D) Helical-wheel representations for Ser-modified heptads; *N* or *C* represents the terminus closer to the viewer. (B,E) Cross sections through the parent serine-containing models. The former was made and optimized in ISAMBARD based on X-ray crystal structures for CC-Tet (PDB entry 3R4A),^43^ and the latter was modeled directly into the apCC-Tet structure (PDB entry 6Q5S)^44^ in PyMol.^52^ (C,F) Cross sections through the phosphoserine-containing models created using the PyMOL plugin PyTMs^51^ from the models of panels B and E.

For the parallel system, we used parameters from the X-ray crystal structure of the original homotetramer CC-Tet,^43^ in ISAMBARD^45^ to generate the following structural models (Figures 2A–C, Table S2). First, two opposite chains were made overall acidic (A) and their intervening neighbors overall basic (B). This entailed making all of the ***g*** and ***e*** sites of the A peptides glutamic acid (Glu, E), and those of the B peptides lysine (Lys, K) (Figure 2A, Table 1). Next, we substituted the leucine (Leu, L) residues at central ***a*** sites (position 14) to Ser in all four helices to create a layer of polar residues in the otherwise hydrophobic core. Our main aim was to ensure that phosphorylation would have a maximal destabilizing impact on coiled-coil assembly. We chose this substitution—rather than Ser@***d***—as it should better accommodate the PKA site (R-R-X-S) by placing the charged Arg residues at the peripheral ***e*** and ***f*** sites of the ***e-f-g-a*** sequence. We modeled the incorporation of this PKA motif into both B peptides (Figures 2A,B) with the justification that (i) only one peptide (A or B) would need to be phosphorylated to effect a switch; and (ii) the basic peptides was better suited to the introduction of positively charged Arg residues. The resulting model had favorable internal energy in ISAMBARD (Table S3) despite the polar cavity created by the four Leu → Ser substitutions (Figure 2B). Finally, and to test assumption (i), we modeled the addition of phosphoryl groups to the phosphoacceptor Ser residues of the B chains. This was done using the PyTMs plugin for PyMol.^51^ The resulting model was not minimized, as it was immediately apparent that the two added phosphoryl groups would clash sterically and repel electrostatically (Figure 2C).

Modeling and design of the antiparallel system followed a similar route, but started from the X-ray crystal structure of apCC-Tet.^44^ First, Ser-containing apCC-Tet-A and apCC-Tet-B sequences were modeled directly onto the chains of apCC-Tet homotetramer. However, the addition of the PKA site to the B chains required more consideration than for the parallel system. This was because ***a*** sites of the A peptides partner with ***d*** sites of the B peptides, and *vice versa*. Also, Ala is required at the ***e*** sites to direct Ala-coil-type interactions that promote the antiparallel state.^44^ Therefore, we modeled the R-R-X-S motif into ***a-b-c-d*** sites of the B peptide (Figures 2D,E). Finally, we replaced Ser-20 of both B chains with phosphoserine. Unlike the parallel model (Figure 2C) where the two phosphoryl groups clashed directly, those of the antiparallel model do not interact sterically (Figure 2F). However, the modified side chains overlap considerably with atoms of the A chain (Figure 2F). Therefore, we anticipated that phosphorylation in either system would strongly disfavor the assembled states.

#### Experimental Characterization

**Figure 3:**
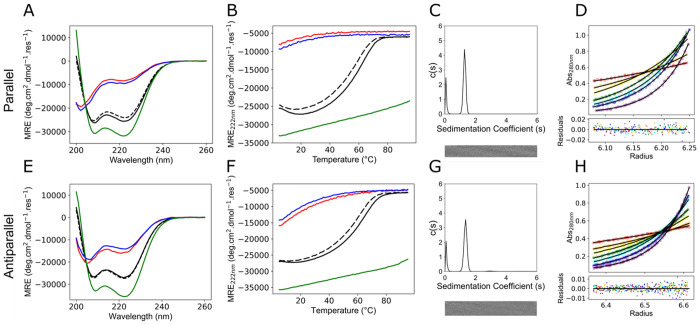
Biophysical characterization of *de novo* designed phosphoswitchable heterotetramers. (A–D) Parallel system. (A) Representative CD spectra at 5 °C and (B) variable temperature CD measurements monitoring MRE_222_ between 5 and 95 °C. Key: CC-Tet-A-S_14_ (red), CC-Tet-B-RRXS_14_ (blue), mixture CC-Tet-A-S_14_ plus CC-Tet-B-RRXS_14_ (black, with postmelt scan and refolding measurements dashed), and the parent heterotetramer CC-Tet-A_2_B_2_ (green).^10^ (C) AUC SV and (D) SE data for the mixture of CC-Tet-A-S_14_ and CC-Tet-B-RRXS_14_, which returned molecular weights of 12956 Da (4.0× monomer mass) and 12568 Da (3.9× monomer mass, 95% confidence limits 12422–12700 Da), respectively. (E–H) Antiparallel system. (E) Representative CD spectra at 5 °C and (F) MRE_222_ between 5 and 95 °C for apCC-Tet-A-S_17_ (red), apCC-Tet-B-RRXS_20_ (blue), the mixture of apCC-Tet-A-S_17_ and apCC-Tet-B-S_20_ (green), and the mixture of apCC-Tet-A-S_17_ and apCC-B-RRXS_20_ (black, with postmelt scan and refolding measurements dashed). (G) AUC SV and (H) SE data for the mixture of apCC-Tet-A-S_17_ and apCC-Tet-B-RRXS_20_, which returned molecular weights of 13066 Da (4.0× average monomer mass) and 12571 Da (3.9× average monomer mass), respectively. Conditions: All measurements were made in phosphate buffered saline (PBS) (pH 7.4), with CD spectra and thermal denaturation curves recorded at 10 μM of each peptide, SV at 70 μM of each peptide, and SE at 35 μM of each peptide.

We characterized the peptides for the parallel system, CC-Tet-A-S_14_ and CC-Tet-B-RRXS_14_. As with the parents, CD spectra revealed the individual peptides were each essentially unfolded (Figures 3A and S9). However, when combined the A:B mixture was folded with α helicity comparable to that of the parent combination, CC-Tet-A_2_B_2_. The Ser-containing combination, was also thermally less stable than the parent heterotetramer consistent with the design rationale.^10^ Moreover, it had a clear, sigmoidal and fully reversible unfolding transition with a midpoint (*T*_M_) of 62 °C (Figure 3B, Table S4). AUC characterization of the mixture confirmed a dominant single species with a molecular weight consistent with a tetrameric assembly (Figures 3C,D). Therefore, CC-Tet-A-S_14_ and CC-Tet-B-RRXS_14_ combine to form an A_2_B_2_ heterotetramer like the parent peptides. For this parallel case, we modeled and characterized peptides with Ser at positions 7, 14, and 21 of the A sequence (Table S2, Figure S7). However, for brevity and simplicity, here we refer only to the characterization of the peptides with Ser@14 in the main body of the text. Similar data with the same conclusions were obtained for the other peptides with Ser at positions 7 and 21 of the A peptide (Figures S10–S13, Table S4).

Similarly, we tested more than one variant of the antiparallel system (Table S1, Figures S8 and S14–S18). However, we focus on one combination here; namely, apCC-Tet-A-S_17_ (with Ser at position 17) and apCC-Tet-B-RRXS_20_ (with the Ser at position 20). These peptides were only partially helical alone Figures 3E,F), but when combined formed a folded heterotetramer with a reversible thermal unfolding transition with a *T*_M_ of 64 °C (Figures 3E–H, Table S4).

Thus, we have achieved the design of parallel and antiparallel heterotetrameric CCs that harbor potential PKA phosphorylation sites. From here on, we refer to the two of these as CC-Tet-A_2_B_2_-S_14_ and apCC-Tet-A_2_B_2_-S_17,20_. The Leu → Ser modifications destabilized these assemblies sufficiently to access the unfolded state upon heating above ≈40 °C at 10 μM peptide concentrations. We contend that this is important for PKA-catalyzed phosphorylation of the Ser residues, because, in the folded states, these side chains are buried in the hydrophobic cores.

### Rapid Phosphoswitching Occurs in Both Systems

**Figure 4:**
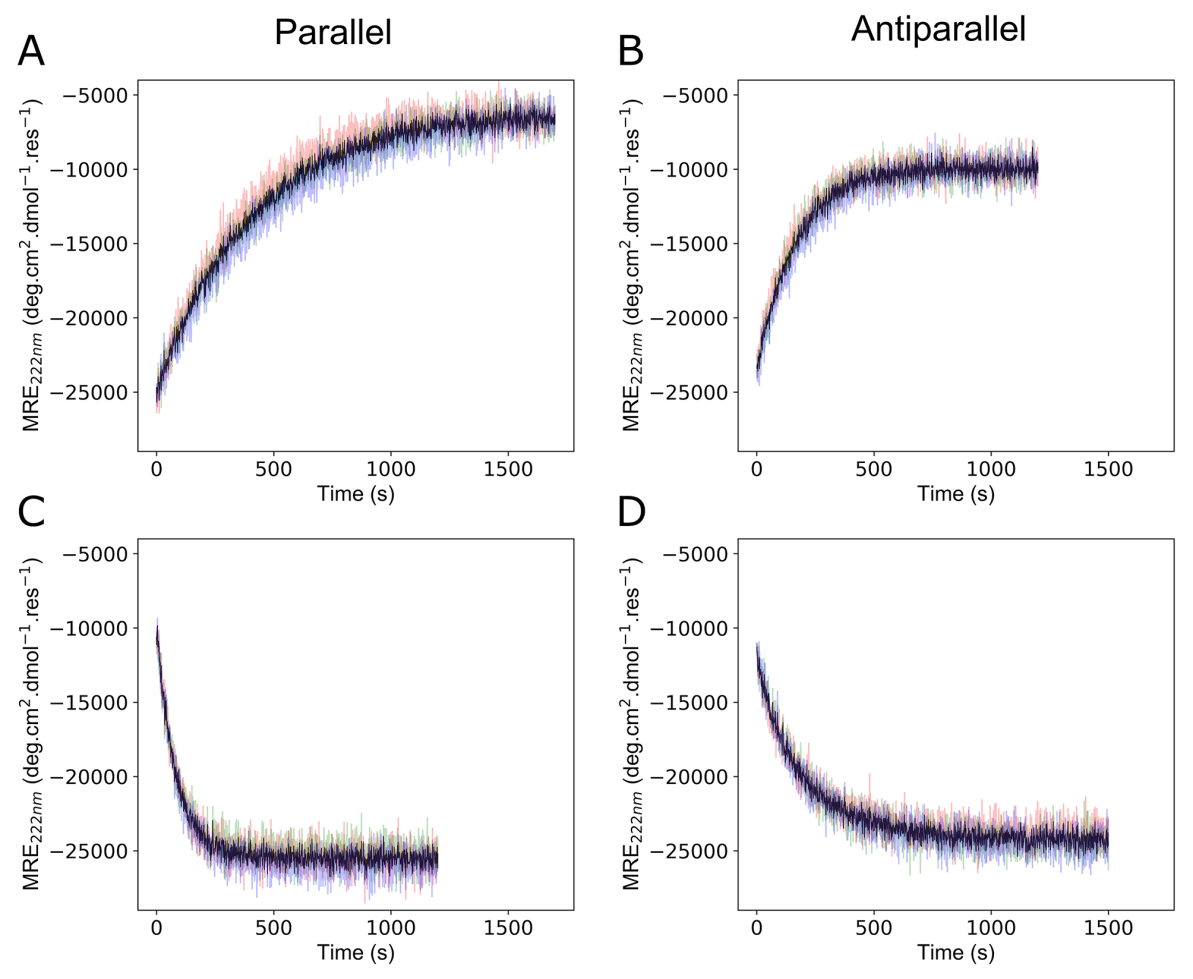
Heterotetramers can be disassembled and reassembled through phosphorylation and dephosphorylation, respectively. (A,B) Representative time courses monitoring MRE_222_ at 37 °C following the addition of PKA at *t*_0_ for (A) CC-Tet-A-S_14_ plus CC-Tet-B-RRXS_14_ (CC-Tet-A_2_B_2_-S_14_) with 5 U/μL PKA and (B) apCC-Tet-A-S_17_ plus apCC-Tet-B-RRXS_20_ (apCC-Tet-A_2_B_2_-S_17,20_) with 1 U/μL PKA. (C,D) Representative time courses monitoring MRE_222_ at 37 °C following kinase inactivation and the addition of 2 U/μL LPP at *t*_0_ for: (C) CC-Tet-A_2_B_2_-S_14_; and (D) apCC-Tet-A_2_B_2_-S_17,20_. These phosphorylation reactions contained 10 μM of each peptide and dephosphorylation reactions 9.85 μM of each peptide. All experiments had *n* = 3, with individual repeats shown in color (red, green, and blue) and the average in black. Note: the transients in panels C and D start at slightly lower MRE_222_ values than where those in A and B end because of an unavoidable experimental lag between mixing the reagents and starting the recordings.

With potentially switchable parallel and antiparallel designs in hand, we investigated the effect of phosphorylation on both assemblies. In separate experiments, the assembled CC-Tet-A_2_B_2_-S_14_ and apCC-Tet-A_2_B_2_-S_17,20_ complexes were incubated in kinase buffer at 37 °C; *i.e.*, just below the starts of thermal unfolding transitions for both systems. Purified PKA was added to the samples and the mean residue ellipticity at 222 nm (MRE_222_)—which reports on α-helical content, and therefore provides a proxy for assembly disruption or formation—was monitored over time (Figures 4A,B).^38,39^

For CC-Tet-A_2_B_2_-S_14_, the MRE_222_ decreased over time (Figures 4A and S20), with start and end points consistent with the equilibrium fully folded and fully unfolded states, respectively. Analysis of the transients with a single exponential function gave a half-life of reaction (*t*_1/2_) of 290 s and an end point at ≈1500 s (Figures 4A and S19, Table S5). Repeating this at a higher peptide concentration resulted in switching occurring over a longer time scale (Figure S21). Phosphorylation was confirmed by MALDI-TOF mass spectrometry with an increase of 79 Da in the mass of the B peptide purified from the mixture after the reaction had completed (Figure S22). Importantly, the molecular weight of the acidic peptide remained unaltered (Figure S22).

Similar responses were observed for apCC-Tet-A_2_B_2_-S_17,20_. However, experiments had to be conducted at a five-times lower concentration (*i.e.*, 1 U/μL versus 5 U/μL) of PKA as changes in MRE_222_ were otherwise too rapid to follow. Under these conditions, the reaction had a *t*_1/2_ of 110 s and was complete by ≈600 s (Figures 4B and S25, Table S5). Again, the final MRE_222_ value was similar to that observed for the individual peptides, and phosphorylation of the B peptide was confirmed by mass spectrometry (Figure S27). Thus, the antiparallel system responds faster to modification than the parallel assembly. This is perplexing given the similar *T*_M_ values for the two assemblies (Figures 3B,F). We speculate that this is due to the substitution of a second hydrophobic core position (L17R) to introduce the PKA consensus motif into the antiparallel system, which could increase the unfolding rate.

As mentioned above, our design process explored variants of the two systems (Table S5 and Figures S19–S29). Specifically for the parallel system, we found that CC-Tet-B-RRXS_14_ paired with A peptides with “mismatched” Ser residues in the preceding and following heptads, CC-Tet-A-S_7_ or CC-Tet-A-S_21_ (Figure S7), to form heterotetramers that were more thermally stable than CC-Tet-A_2_B_2_-S_14_ (Table S4). Therefore, we tested these for PKA-induced switching. Consistent with their increased thermal stabilities, both combinations showed slower phosphorylation kinetics than CC-Tet-A_2_B_2_-S_14_, with *t*_1/2_ of 1650 and 4220 s, respectively (Figures S19, S20, S23, and S24, Table S5). Similarly, the other antiparallel combinations either did not switch to fully destabilized states (*e.g.*, apCC-Tet-A-S_10_ plus apCC-Tet-B-RRXS_20_), or switched over a very long time period (*e.g.*, apCC-Tet-A-S_17_ plus apCC-Tet-B-RRXS_13_) (Figures S25, S26, S28, and S29, Table S5). In summary, we have generated several phosphoswitchable CCs with different switch kinetics, which may find uses when different switching times are required.

### Phosphoswitching Is Reversible

To investigate if phosphorylation could be reversed by a protein phosphatase and the complexes reformed *in situ*, samples from the above reactions were heated to 75 °C for 30 min to denature PKA; cooled to 37 °C; and then purified Lambda Protein Phosphatase (LPP) plus MnCl_2_ (required for phosphatase activity), were added (Figures 4C,D).^38,39^ For the phosphorylated CC-Tet-A_2_B_2_-S_14_ mixture, this led to a rapid increase in MRE_222_ intensity to a value close to that expected for the fully assembled state (compare Figures 4C, 4A and 3A). The transition had a *t*_1/2_ of 58 s and was complete by ≈550 s (Table S5). For the antiparallel system, phosphorylated apCC-Tet-A_2_B_2_-S_17,20_, the response had a *t*_1/2_ of 140 s, was complete by ≈1200 s, and reached a similar final MRE_222_ to the expected fully assembled state (Figure 4D, Table S5). Furthermore, for the aforementioned combinations of variants of the two systems, the *t*_1/2_ values and completion rates were similar within each system (Figures S19 and S25, Table S5). For instance, the *t*_1/2_ values were in the range of 58–75 s for the parallel assemblies, and 140–180 s for the antiparallel combinations. The similarities of these numbers within each set suggests that LPP is acting on the unfolded states of the phosphorylated peptides. Following this, we posit that the difference between the two systems are due to sequence-specific effects, Table 1.

In summary, the two designs represent reversible switching systems with distinct on and off states.

### A Quantitative Model for the Forward Switch (Phosphorylation) Reaction

**Figure 5:**
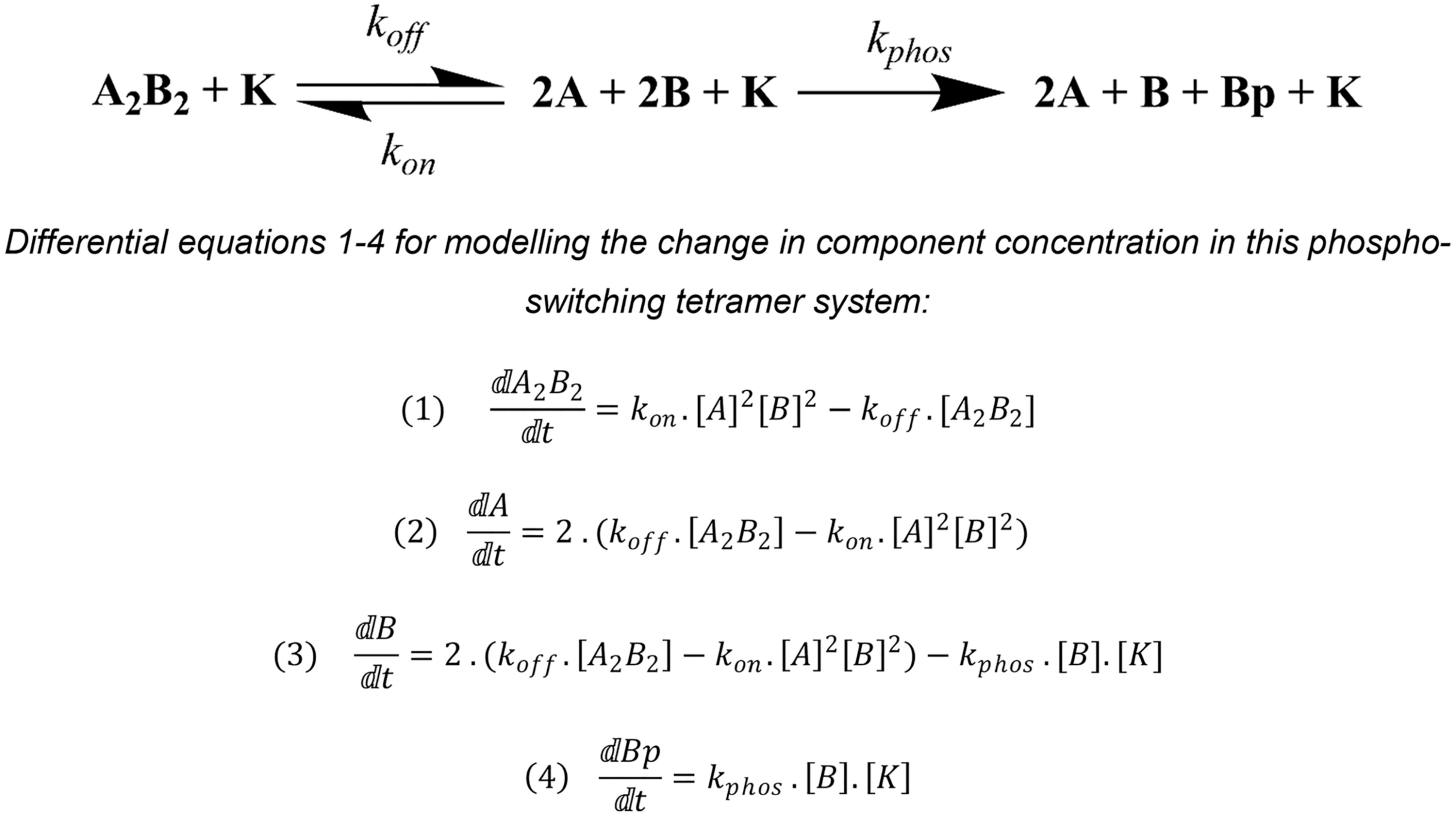
Phosphoswitching tetramer model and resulting differential equations. The acidic, basic, and phosphorylated basic peptides are denoted A, B, and Bp, respectively, and the kinase (PKA) as K. The rate constants are *k*_on_, association rate constant; *k*_off_, dissociation rate constant; and *k*_phos_, phosphorylation rate constant.

Given the experimental robustness of our *de novo* designed phosphorylation-based switches, we characterized the CC-Tet-A_2_B_2_-S_14_ system further. We chose the parallel system because of our experience with these and future plans to take the switch into cells.^10^ Specifically, we sought to develop a mechanistic model for the forward, phosphorylation reaction (Figure 5). For this, we assumed that the tetrameric complex disassembles to populate an unfolded state to make the Ser side chain accessible to kinase; and that any non-enzyme-catalyzed dephosphorylation of the phosphorylated B peptides is negligible.

**Figure 6:**
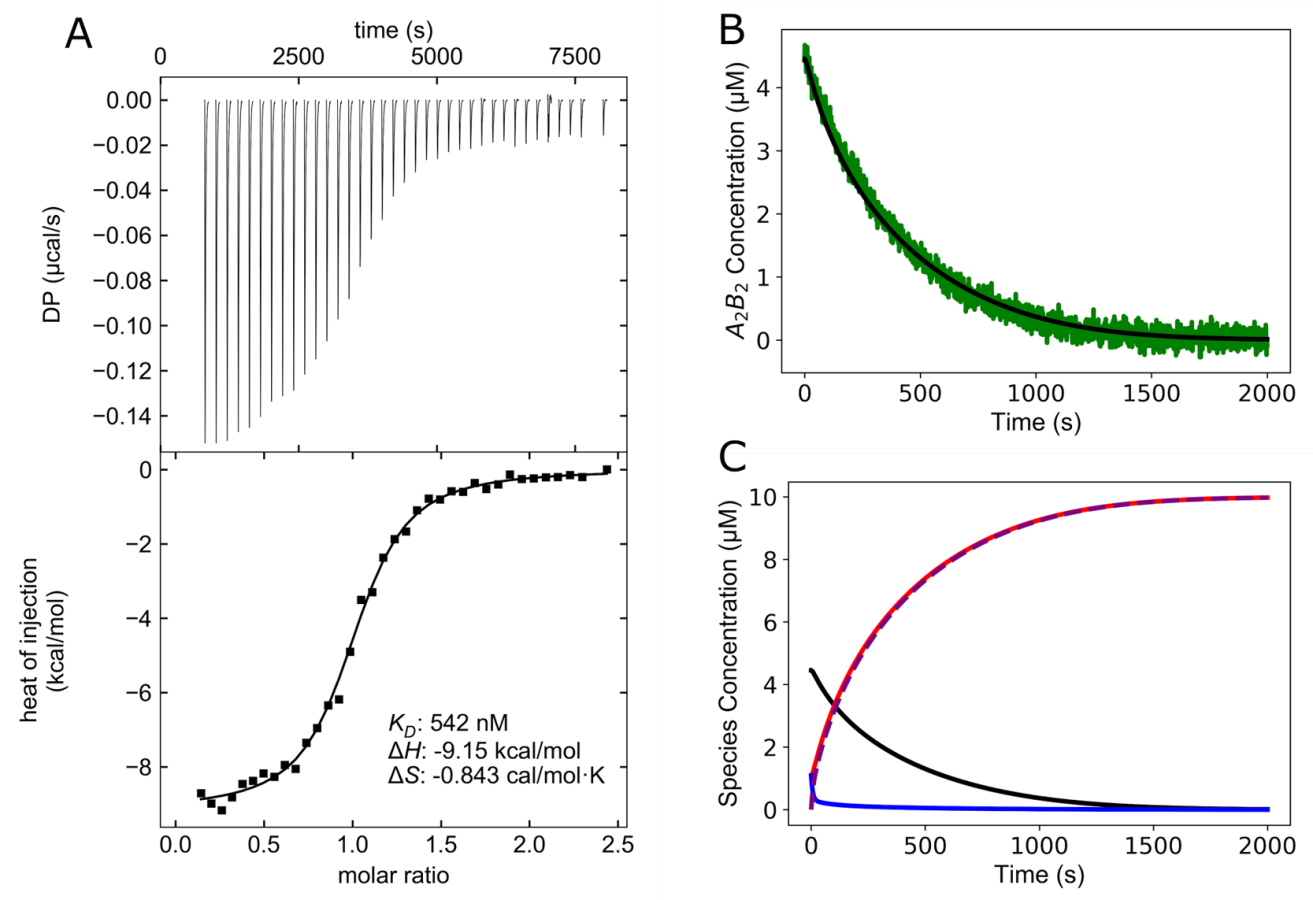
Isothermal titration calorimetry and modeling of the parallel CC-Tet-A_2_B_2_-S_14_ system. (A) (top) Representative injection peaks against time and (bottom) binding isotherms created by plotting the integrated heat peaks against the molar ratio, for CC-Tet-A-S_14_ plus CC-Tet-B-RRXS_14_. The observable dissociation constant (*K*_D_), and changes in enthalpy (Δ*H*) and entropy (Δ*S*) upon binding are displayed. Here 1 μL aliquots of 200 μM CC-Tet-B-RRXS_14_ were sequentially injected into 200 μL of 20 μM CC-Tet-A-S_14_. Experiments were conducted at 37 °C and data shown are after global fitting (*n* = 3). (B) Comparison of experimentally determined phosphorylation data from CD spectroscopy with results produced by the model after optimization of *k*_off_ and *k*_on_ parameters. Normalized CD phosphorylation reaction data for loss of tetramer are plotted in green, and the model fit is plotted in black. (C) Modeling the concentration of component species during the phosphorylation of the CC-Tet-A_2_B_2_-S_14_ system, using parameters extracted from experimental data. Key: CC-Tet-A-S_14_ (red), CC-Tet-B-RRXS_14_ (blue), CC-Tet-A_2_B_2_-S_14_ (black), phosphorylated CC-Tet-B-RRXS_14_ (purple dash). Modeling using differential equations 1–4 (Figure 5) and parameters from Table S6.

To model the disassembly of the A_2_B_2_ complex into the unfolded peptides A and B, we needed to determine the dissociation constant (*K*_D_) and use this to estimate the forward and reverse rate constants, *k*_off_ and *k*_on_ (Figure 5). To do this, we used isothermal titration calorimetry (ITC), titrating CC-Tet-B-RRXS_14_ into 20 μM CC-Tet-A-S_14_ at 37 °C, and the heat released upon binding was measured. (*N.B*. The individual peptides, CC-Tet-A and CC-Tet-B, of the high-affinity parent heteromer do not self-associate even at 100 μM.^10^) The resulting data were fitted to a heterodimer model to give an observable *K*_D_ of 542 nM (defined as the concentration at which the complex is half folded), and a stoichiometry of ≈1:1 (Figure 6A). We contend that this sub-μM *K*_D_—which is consistent with our CD measurements (Figure S30)—is close to ideal for use in synthetic biology systems in cells and *in vitro*, as functioning at μM concentrations leaves the system poised in a folded state, but close to unfolding, rendering it receptive to phosphorylation-induced switching.

This *K*_D_ was then used to calculate the theoretical tetrameric *K*_D(T)_ and the concentrations of individual components under our experimental phosphorylation conditions. That is, at the *K*_D_ the concentrations of the folded A_2_B_2_ and unfolded A + B species are equal (at 0.542 μM). This gives . In turn, this was used to calculate the concentrations of species in the CD phosphoswitching experiments (10 μM individual peptides, Figure 4A), required as starting points for modeling the phosphorylation of the tetramer complex. This gave values of: 1.093 μM of each of the unfolded peptides, A and B, and 4.454 μM of the assembled complex, A_2_B_2_ (Figure S31).

Next, we sought parameters for the phosphorylation of the B peptide. Given the similarity between the classical PKA substrate Kemptide (LRRASLG) and CC-Tet-B-RRXS_14_ (...IRRKSAA...), we used literature values of *K*_M_ and *k*_cat_ for this substrate,^53^ determined using the same murine catalytic subunit of PKA as used in our experiments (Figure 4A). These provided an estimate of *k*_cat_/*K*_M_ (1.06 × 10^6^ M^–1^ s^–1^),^53^ which we used as the rate constant when modeling the phosphorylation reaction.

We used the ordinary differential equations and the parameters determined above (Figure 5 and Table S6) to produce a mechanistic model of this phosphoswitching system. We used the curve fit function of Berkeley Madonna^54^ to optimize the values of parameters *k*_off_ and *k*_on_ (maintaining the *K*_D(T)_ as 3.18 × 10^–19^ M^3^) to fit to the normalized experimental CD phosphorylation data (Figure 4A). After optimizing these parameters, we found good agreement between the experimental CD data and modeled data (Figure 6B). The final model provides estimates for the following parameters in our system: *k*_off_ = 0.0038 s^–1^ and *k*_on_ = 1.1935 × 10^16^ M^–3^ s^–1^, which, taking the cube into account, predicts an observed *k*_on_ of 2.29 × 10^5^ M^–1^ s^–1^. These values are comparable with those reported in the literature for CC heterodimers,^55,56^ and are within the typical range for PPIs.^57,58^ Using these parameters, our model was able to simulate the concentrations of all components throughout the course of the phosphorylation reaction (Figure 6C). Therefore, the model is useful in elucidating the mechanistic process of CC-Tet-A_2_B_2_-S_14_ phosphoswitching and provides information on key parameters involved in controlling the rate of switching, most importantly the dissociation of the complex dictated by *k*_off_. Also, we hope that it will be useful to others who want to use or adapt this system for other applications.

## Conclusion

In summary, first, we have added a new module to the growing toolkit of *de novo* CC peptides, namely an antiparallel heterotetrameric assembly, apCC-Tet-A_2_B_2_. This may prove useful to other protein engineers and synthetic biologists given the abundance of antiparallel CCs and PPIs in nature.^46^ Second, we have shown that apCC-Tet-A_2_B_2_ and its parallel analogue, CC-Tet-A_2_B_2_,^35^ can be redesigned rationally to incorporate functional phosphorylation sites. The resulting redesigns are stably and cooperatively folded. Moreover, they access unfolded states to permit enzymatic phosphorylation, which drives their complete disassembly. The switches occur rapidly (within minutes of initiation), reversibly, and at physiologically relevant temperatures and concentrations. Finally, we have produced a mechanistic model that captures the phosphorylation-induced unfolding switch of the CC-Tet-A_2_B_2_-S_14_ system. This provides estimates for parameters that could not otherwise be determined. Therefore, given the current lack of similar systems in the literature, we believe that the model will be useful for manipulating ours and similar designs of switchable PPI domains in the future.

The new systems that we outline offer advantages over other switchable CC systems:^38,39^ First, the new systems have high switching rates and cooperativity afforded by their higher oligomeric state. Second, they result in full destabilization of the CC complex in response to phosphorylation, in contrast to other designs that only partially stabilize or destabilize the interaction in response to this modification.^38^ Finally, the ability to model one of the systems provides a means for anticipating how it might respond to changes to the design and when ported into other contexts for applications in synthetic biology.

One aim of synthetic biology is to implement novel protein components into living systems, be this simply replacing existing natural parts, or, more ambitiously, constructing entirely new synthetic systems from designed components. While protein design has been successful in delivering hyper-thermally stable proteins that adopt a single state,^59−61^ much less attention has been paid to designing protein dynamics and conformational changes.^4,20,21,62^ By contrast, in nature, the majority of proteins are only marginally stable, and many rely on inherent dynamic behaviors to fulfill their roles.^1^ Examples of this include conformational changes in response to ligand binding; substrate binding, catalysis, and product release in enzymes; and in signal transduction, where switchable PPIs permit high-speed molecular signaling that does not rely on the transcription and translation of additional effector proteins.^63,64^ More specifically, phosphorylation commonly acts to produce disorder-to-order and order-to-disorder transitions in targeted proteins, thereby creating, modifying or destroying PPI domains.^65^ Therefore, it is important that such features can be designed into *de novo* proteins, to allow the replacement of natural parts and processes with designed versions for improved or augmented functions.^21^ Toward this goal, in a similar manner to natural signaling pathways, we have used phosphorylation to produce *de novo* designed switchable heterotetrameric PPI domains.

Switchable CCs and PPI domains such as those presented here could be used *in vitro* for the controlled disassembly of large peptide/protein-based structures and *in vivo* to control protein association post-transcriptionally. Given the rate of kinase kinetics, such switches could afford faster responses than is possible through transcriptional control alone. Our future work will pursue this by implementing the systems in cells to render *de novo* designed CC switches *in vivo*. These could present alternative strategies for post-transcriptional control in genetic circuits.^66,67^ The two configurations that we describe—*i.e.*, parallel and antiparallel arrangements of peptide chains—offer versatility in constructing such systems.

## Methods

### Peptide Synthesis and Purification

Peptides were synthesized on a 0.1 mmol scale on Rink Amide Chemmatrix resin (Novabiochem) using a Liberty microwave-assisted peptide synthesizer (CEM). Synthesis was carried out using a standard Fmoc solid-phase synthesis protocol. Deprotection was achieved by two cycles of 20% morpholine (Thermo Fisher) in DMF (Cambridge Reagents) at 90 °C for 1 min. Coupling was conducted as a single cycle using Fmoc-amino acids, Cl-HOBt (both Cambridge Reagents) and diisopropylcarbodiimide (Thermo Fisher) in DMF at 75 °C for 5 min, except for Fmoc-Arg which underwent a double coupling cycle. Following synthesis, *N*-acetylation was carried out using acetic acid anhydride/diisopropylethylamine (DIPEA) (1:2) (both Thermo Fisher) in 7 mL DMF for 20 min. Peptides were released from the resin and side-chain protecting groups were removed by treatment with 10 mL of trifluoracetic acid (TFA), triisopropylsilane (TIPS) (both Thermo Fisher), and water (95:2.5:2.5), for 3.5 h at room temperature. Nitrogen was then used to concentrate the sample to approximately 5 mL. Crude peptide was precipitated in 25 mL ice-cold diethyl ether, isolated by centrifugation (3000 rpm, 10 min, 4 °C), dissolved in 1:1 acetonitrile/water and then lyophilized.

Peptides were purified by reverse-phase HPLC (RP-HPLC) using a Kromatek C18 column (5 μM, 100 Å, 150 × 10 mm). A combination of buffer A (water and 0.1% TFA) and buffer B (acetonitrile and 0.1% TFA) was applied to the column at a flow rate of 3 mL/min at different linear gradient of 20–80% buffer B over 30 min. Fractions collected were examined by MS on an UltraFleXtreme (Bruker) MALDI-TOF instrument using α-cyano-4-hydroxycinnamic acid (Sigma-Aldrich) as the matrix. Those containing exclusively the desired product were pooled and analyzed by analytical RP-HPLC using a Kinetex C18 column (2.6 μm C18 100 Å, LC Column 100 × 4.6 mm) on a JASCO chromatography system and buffers A and B at gradients stated above over a period of 20 min. Absorbance at 220 and 280 nm was normalized and plotted to ensure peptide purity. Peptide concentrations were determined by UV absorbance (ε280 (Trp) = 5690 mol^–1^ cm^–1^) using a NanoDrop 2000 Spectrophotometer (Thermo Fisher).

### Circular Dichroism (CD) Spectroscopy

CD spectra and thermal denaturation curves were obtained using a JASCO J810 or 815 spectropolarimeter fitted with a Peltier temperature controller. CD spectra (260 to 190 nm, with 1 nm step size, 100 nm.min^–1^ scanning speed, 1 nm bandwidth and 1 s response time) were measured in PBS (137 mM NaCl, 2.7 mM KCl, and 10 mM phosphate buffer, pH 7.4) at 5 °C in quartz cuvettes. Thermal denaturation experiments were performed using 10 μM individual peptide concentration in a 5 mm cuvette. For thermal denaturation experiments, the CD signal at 222 nm was recorded at 1 °C intervals upon heating from 5 to 95 °C at a rate of 40 or 60 °C/h (1 nm interval, 1 nm bandwidth, 16 s response time). Data was buffer subtracted, and then normalized for peptide concentration, number of amide bonds present, and cuvette path length to give the mean residue ellipticity (MRE). Midpoints of thermal denaturation were calculated using the second derivative of the corresponding thermal denaturation curve. Fraction helix (%) was calculated using the following equation (where MRE_coil_ = 640 – 45*T*; *T*, temperature (°C); *n*, number of amide bonds in sample including C-terminal amide):^68^
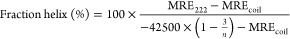


### Analytical Ultracentrifugation (AUC)

Sedimentation-velocity (SV) experiments were conducted at 20 °C in a Beckman-Optima XL-I analytical ultracentrifuge using an An-60 Ti rotor. Samples were prepared in PBS at 140 μM total peptide concentration. Samples were centrifuged at a speed of 50,000 or 60,000 rpm. The partial specific volume (*v̅*) for each peptide was calculated and data sets were fitted to a single, ideal species model using SEDFIT. Sedimentation-equilibrium (SE) experiments were conducted at 20 °C in a Beckman-Optima XL-I analytical ultracentrifuge using an An-50 Ti rotor. Samples were prepared in PBS at 70 μM total peptide concentration. Samples were centrifuged at a range of speeds from 18,000–39,000 rpm with defined intervals, and 8 h between subsequent scans. Data sets were analyzed using SEDPHAT.

### Fluorescence-Quenching Experiments

Peptide concentrations were measured at 214 nm^69^ using an Agilent Cary 100 UV–vis spectrophotometer and a 10 mm quartz cuvette. Fluorescence emission spectra were acquired on a JASCO FP-6300 fluorimeter using a 10 mm quartz cuvette and 50 μM individual peptide in phosphate buffer (8.2 mM sodium phosphate dibasic, 1.8 mM potassium phosphate monobasic), pH 7.4. Spectra were recorded as the average of three scans from 260 to 400 nm, with an excitation wavelength of 240 nm, a data pitch of 0.5 nm and a scanning speed of 200 nm/min. Data was blank subtracted and normalized to the highest observed signal.

### Modeling Structures and Effect of Phosphorylation

Parametric models of complexes were produced using ISAMBARD^45^ and further optimized using a genetic algorithm to minimize BUFF (Bristol University Docking Engine Force Field) values.^70,71^ The lowest energy models were visualized using PyMol (Schrödinger). *In silico* phosphorylation was carried out using PyTMs plugin,^51^ which optimized the placement of the phosphoryl group to avoid clashes.

### Peptide Phosphorylation and Dephosphorylation

Peptide phosphorylation was monitored by recording the CD signal at 222 nm against time.^38,39^ Peptides at 20 or 100 μM total concentration in 50 mM Tris 150 mM NaCl (pH 7.6) supplemented with 500 μM ATP and 10 mM MgCl_2_ (phosphorylation buffer), were incubated with 1 or 5 U/μL cAMP-dependent Protein Kinase (PKA), catalytic subunit (NEB) at 37 °C in a 1 or 5 mm quartz cuvette respectively for a maximum of 6 h, with time points taken every 1 to 5 s based on incubation time.

Prior to dephosphorylation, samples were heated to 75 °C for 30 min in the CD instrument to inactivate the kinase. Following this, MnCl_2_, to a final concentration of 1 mM (dephosphorylation buffer), and then 2 U/μL Lambda Protein Phosphatase (LPP) (NEB) were added, and the sample was incubated at 37 °C for up to 1 h. Peptide dephosphorylation was again monitored by recording the CD signal at 222 nm against time, with time points taken every second for the duration of the reaction. Data was then processed as above. Peptides were analyzed for phosphorylation by RP-HPLC, with peaks collected and analyzed by MALDI-TOF MS.

The kinetics of the reactions were calculated in Python using the curve_fit function from scipy Python package. Here experimental data was fitted to the following exponential equation (*y*, MRE_222_; *x*, time; *a*, *b*, and *c*, constants):
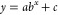
The resulting equation of the fit was rearranged to give the equation below. This equation was then used to calculate the half-life of the reaction with variables calculated from the fit equation (*x*, reaction half-life; ln, natural logarithm; *y*, midpoint after loss of half of total signal; *a*, *b*, and *c*, constants):
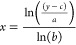


### Isothermal Titration Calorimetry (ITC)

Peptides were dissolved in PBS buffer to a concentration around 1 mM and a volume of 1 mL. Samples were then individually dialyzed into 1.5 L of PBS with gentle stirring overnight using a Mini Dialysis Kit (Cyvita) with a 1 kDa membrane cut off. Sample concentration was subsequently remeasured to account for changes in volume because of diffusion during dialysis.

Isothermal titration calorimetry (ITC) was performed using a MicroCal iTC200 (GE Healthcare) following manufacturer’s recommendations. One peptide was diluted to a concentration of 200 μM, to be loaded into the sample injector syringe (capacity ∼38 μL), and the other peptide partner to a concentration of 20 μM to be loaded into the sample cell (capacity ∼200 μL). After washing with the entire system with Hellmanex solution and then buffer, the reference and sample cell were filled with PBS and sample at 20 μM, respectively, using a 0.5 mL Hamilton gastight glass syringe, ensuring the removal of any bubbles. Peptide sample at 200 μM was loaded into the injector syringe. The system was then set to equilibrate to a temperature of 37 °C and an experiment protocol was set up with the following parameters: total injections 39, cell temperature 37 °C, reference power 10 μcal/s, initial delay 400 s, syringe and cell concentration 200 and 20 μM respectively, stirring speed 1000 rpm, 1 μL injection with 200 s spacing and 5 s filter.

To analyze the data, NITPIC, SEDPHAT, and GUSSI were used.^72−74^ Briefly, using NITPIC each thermogram was first integrated and then background subtracted (corresponding to titration of the sample injected into buffer alone). Following this, experiments were analyzed using SEDPHAT, which allowed correction of concentration deviations and outlier injection exclusion, after which data was fitted to a heterodimer model (as no heterotetramer model was available). Next, global fitting across three experiments was performed, which was then plotted using GUSSI.

### Modeling the Phosphoswitching System

The Berkeley Madonna^54^ ordinary differential equations (ODE) solver package was used to model the phosphoswitching system. Here the chemical reactions module was used to produce a model and 4 corresponding ODEs to model the change in concentration of the components of the system (Figure 5). Parameters for this model were largely taken (or calculated) from experimentally determined values and are detailed in Table S6. CD phosphorylation data was normalized by first subtracting the post phosphorylation baseline from the data (calculated as the average of the last 10 data points), and then dividing this value by the range of the data (calculated as the difference between the average of the first 10, and last 10 data points). The resulting value was then multiplied by 4.454 × 10^–6^, given the model suggested this was the concentration of tetramer present at 10 μM of each peptide (based on experimentally derived *K*_D_). This normalized CD data was used to optimize the value of the parameters *k*_on_ and *k*_off_ (while maintaining *K*_D_ as 3.18 × 10^–19^ M^3^), using the curve fit function in Berkeley Madonna. Resulting data from the model and normalized CD phosphorylation data was plotted with using Python.

## Supporting Information Available

Supplementary Figures S1–S31: Images for additional structural models, HPLC, MS, and additional biophysical characterization data for all peptides, and additional kinetic transients from the switching experiments; Supplementary Tables S1–S6: Peptide sequences, summaries of the biophysical data, and the variables used for modeling the phosphoswitching reaction; Supplementary References (PDF)
