# Supplementary material for: Rational Design of Phosphorylation-Responsive Coiled Coil-Peptide Assemblies
Source: ACS Synth Biol. 2023 Mar 29;12(4):1308–19. doi: 10.1021/acssynbio.3c00064 (PMC10127268; doi:10.1021/acssynbio.3c00064)
Supplement: Supplementary file 1 — sb3c00064_si_001.pdf [file sb3c00064_si_001.pdf]

Rational Design of Phosphorylation-responsive Coiled coil-peptide Assemblies

Supplementary Information

Harry F. Thompson,[1,2] Joseph L. Beesley,[1,2] Hannah D. Langlands,[1,2] Caitlin L. Edgell,[1,2] Nigel J. Savery,[1,3,*] and Derek N. Woolfson[1,2,3,*]

[1]School of Biochemistry, University of Bristol, University Walk, Bristol, BS8 1TD, UK

[2]School of Chemistry, University of Bristol, Cantock's Close, Bristol, BS8 1TS, UK

[3]Bristol BioDesign Institute, School of Chemistry, University of Bristol, Cantock's Close, Bristol, BS8 1TS, UK

*To whom correspondence could be address: N.J.Savery@bristol.ac.uk; D.N.Woolfson@bristol.ac.uk

Supplementary Figures

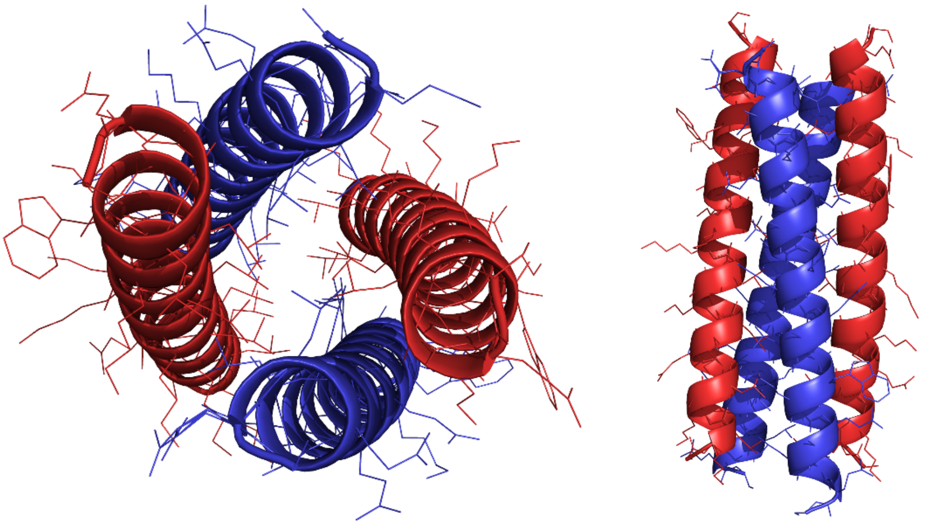

Figure S1. Modelling A_2B_2 antiparallel heterotetramer initial designs. (Left) Top-down view and (right) side on view of model of initial apCC-Tet-A_2B_2. Models produced in ISAMBARD[1] and images captured using Pymol.

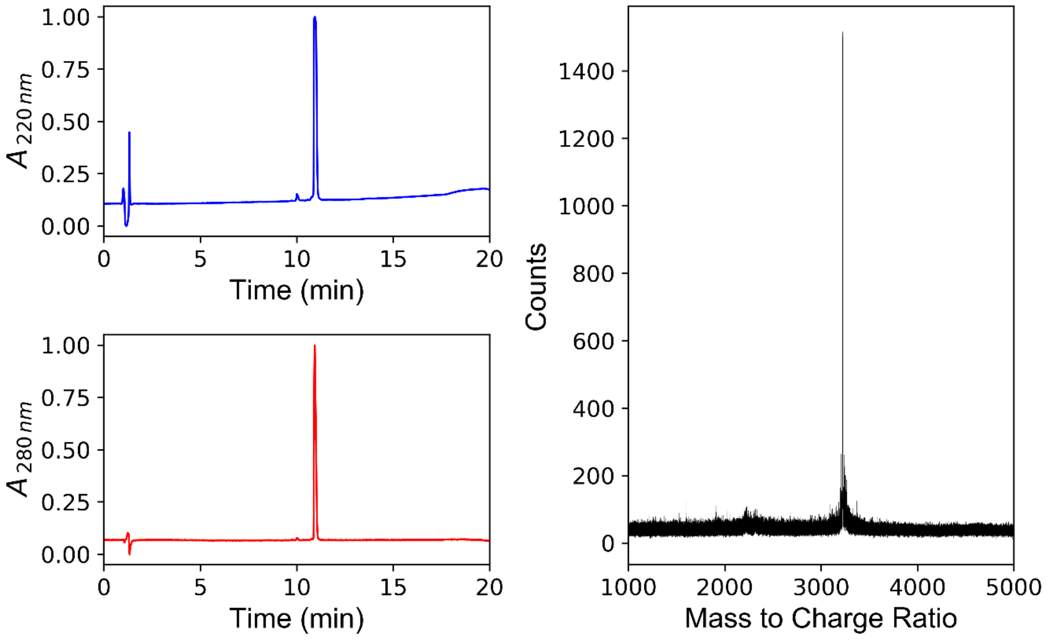

Figure S2a. Analytical HPLC and MS data for CC-Tet-A-S_{14}. (Left) Representative analytical HPLC chromatogram monitoring absorbance at 220 nm (blue) and 280 nm (red) of peptide CC-Tet-A-S_{14}. (Right) Representative mass spectrum of peptide CC-Tet-A-S_{14} (black), with expected and observed masses of 3223.7 and 3222.6 Da, respectively.

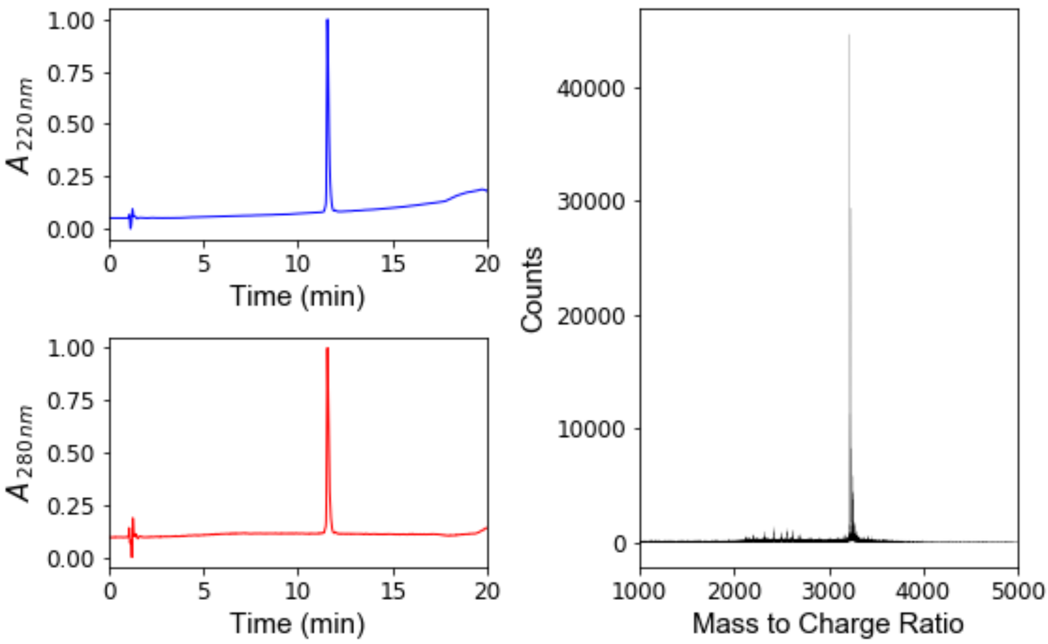

Figure S2b. Analytical HPLC and MS data for CC-Tet-A-S_7. (Left) Representative analytical HPLC chromatogram monitoring absorbance at 220 nm (blue) and 280 nm (red) of peptide CC-Tet-A-S_7. (Right) Representative mass spectrum of peptide CC-Tet-A-S_7 (black), with expected and observed masses of 3223.7 and 3225.0 Da, respectively.

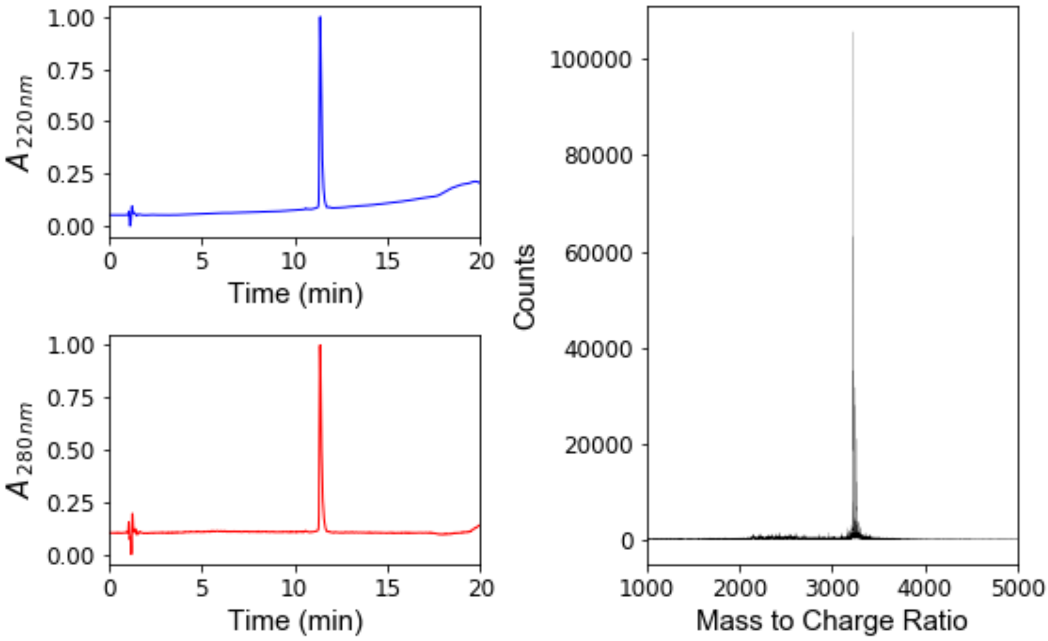

Figure S2c. Analytical HPLC and MS data for CC-Tet-A-S_{21}. (Left) Representative analytical HPLC chromatogram monitoring absorbance at 220 nm (blue) and 280 nm (red) of peptide CC-Tet-A-S_{21}. (Right) Representative mass spectrum of peptide CC-Tet-A-S_{21} (black), with expected and observed masses of 3223.7 and 3225.0 Da, respectively.

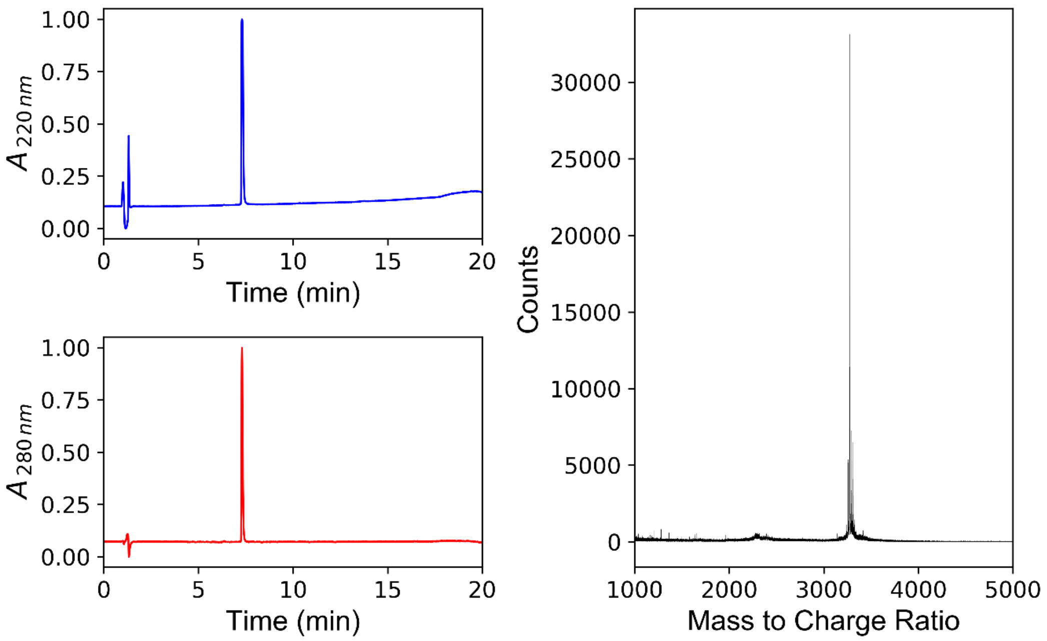

Figure S2d. Analytical HPLC and MS data for CC-Tet-B-RRXS$_{14}$. (Left) Representative analytical HPLC chromatogram monitoring absorbance at 220 nm (blue) and 280 nm (red) of peptide CC-Tet-B-RRXS$_{14}$. (Right) Representative mass spectrum of peptide CC-Tet-B-RRXS$_{14}$ (black), with expected and observed masses of 3272.1 and 3272.9 Da, respectively.

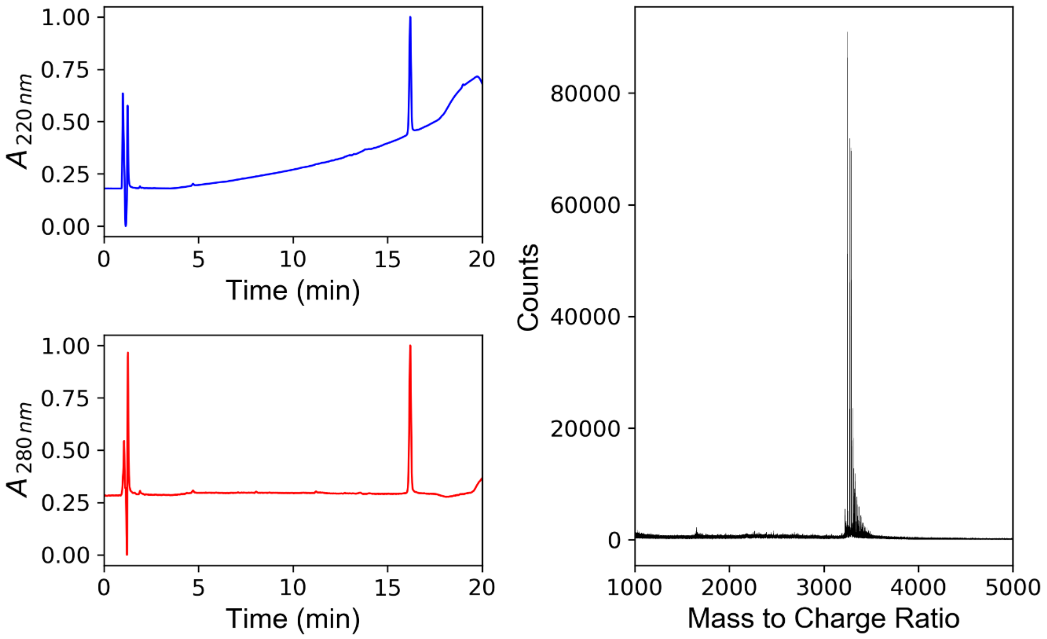

Figure S2e. Analytical HPLC and MS data for apCC-Tet-A1. (Left) Representative analytical HPLC chromatogram monitoring absorbance at 220 nm (blue) and 280 nm (red) of peptide apCC-Tet-A1. (Right) Representative mass spectrum of peptide apCC-Tet-A1 (black), with expected and observed masses of 3250.6 and 3250.1 Da, respectively.

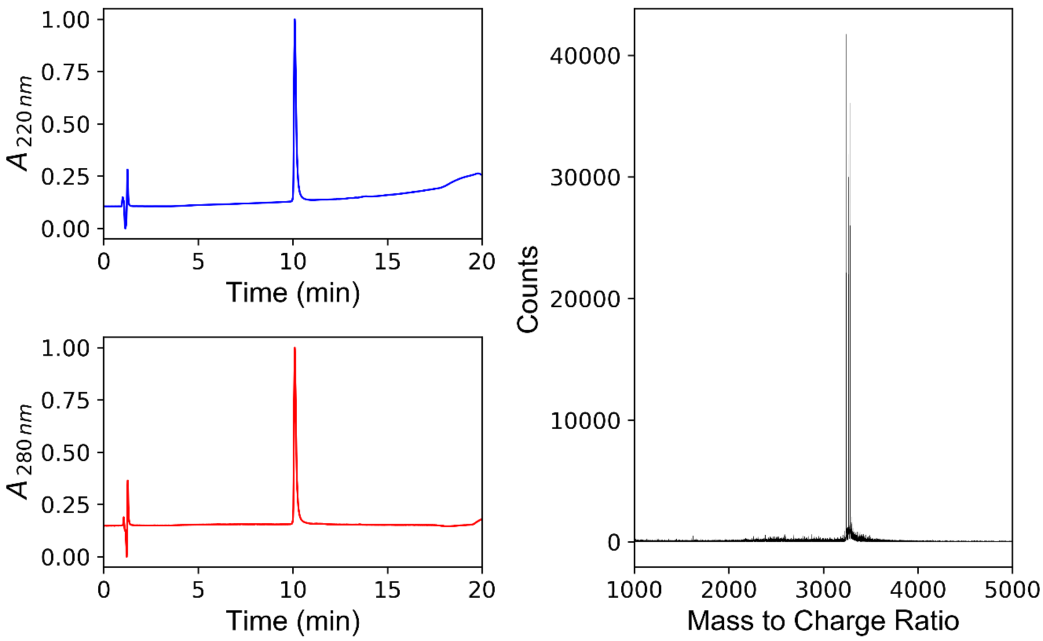

Figure S2f. Analytical HPLC and MS data for apCC-Tet-B1. (Left) Representative analytical HPLC chromatogram monitoring absorbance at 220 nm (blue) and 280 nm (red) of peptide apCC-Tet-B1. (Right) Representative mass spectrum of peptide apCC-Tet-B1 (black), with expected and observed masses of 3243.1 and 3243.3 Da, respectively.

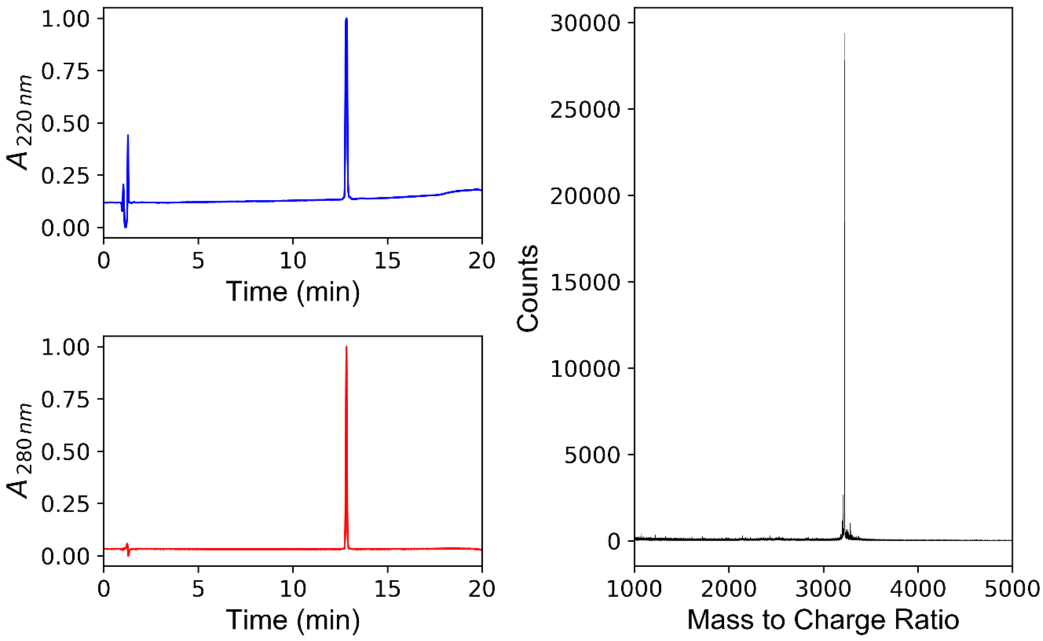

Figure S2g. Analytical HPLC and MS data for apCC-Tet-A-S_{17}. (Left) Representative analytical HPLC chromatogram monitoring absorbance at 220 nm (blue) and 280 nm (red) of peptide apCC-Tet-A-S_{17}. (Right) Representative mass spectrum of peptide apCC-Tet-A-S_{17} (black), with expected and observed masses of 3224.5 and 3223.5 Da, respectively.

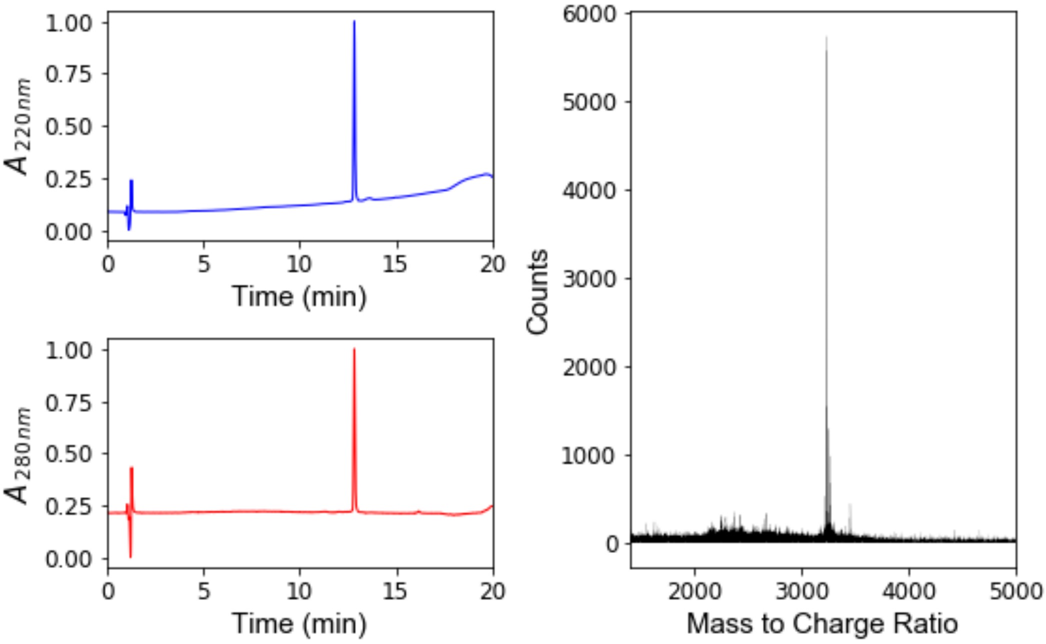

Figure S2h. Analytical HPLC and MS data for apCC-Tet-A-S_{10}. (Left) Representative analytical HPLC chromatogram monitoring absorbance at 220 nm (blue) and 280 nm (red) of peptide apCC-Tet-A-S_{10}. (Right) Representative mass spectrum of peptide apCC-Tet-A-S_{10} (black), with expected and observed masses of 3224.5 and 3224.9 Da, respectively.

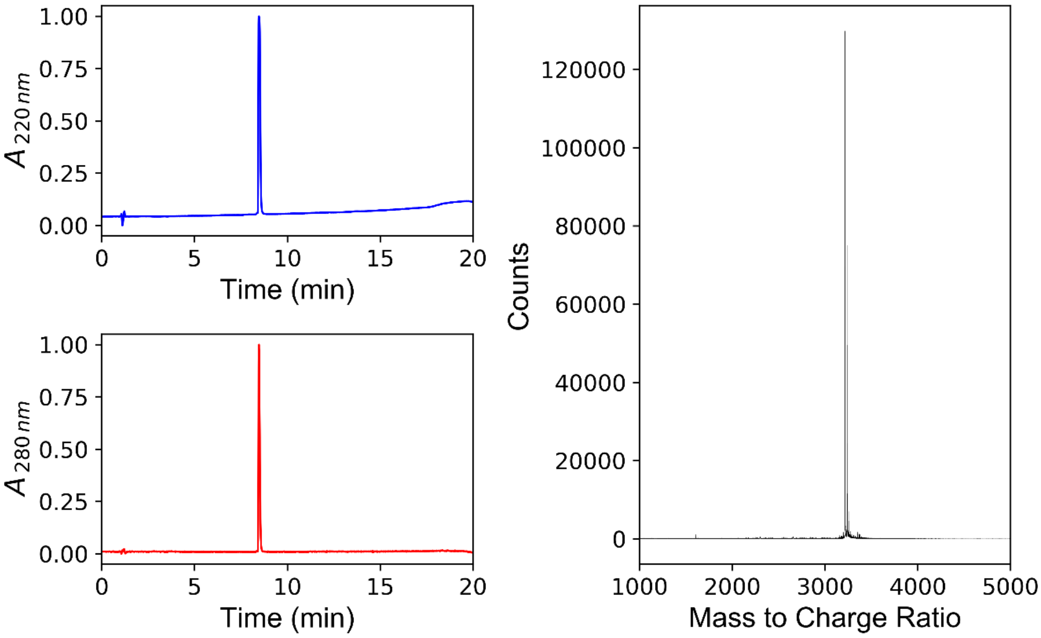

Figure S2i. Analytical HPLC and MS data for apCC-Tet-B-S_{20}. (Left) Representative analytical HPLC chromatogram monitoring absorbance at 220 nm (blue) and 280 nm (red) of peptide apCC-Tet-B-S_{20}. (Right) Representative mass spectrum of peptide apCC-Tet-B-S_{20} (black), with expected and observed masses of 3217.0 and 3215.7 Da, respectively.

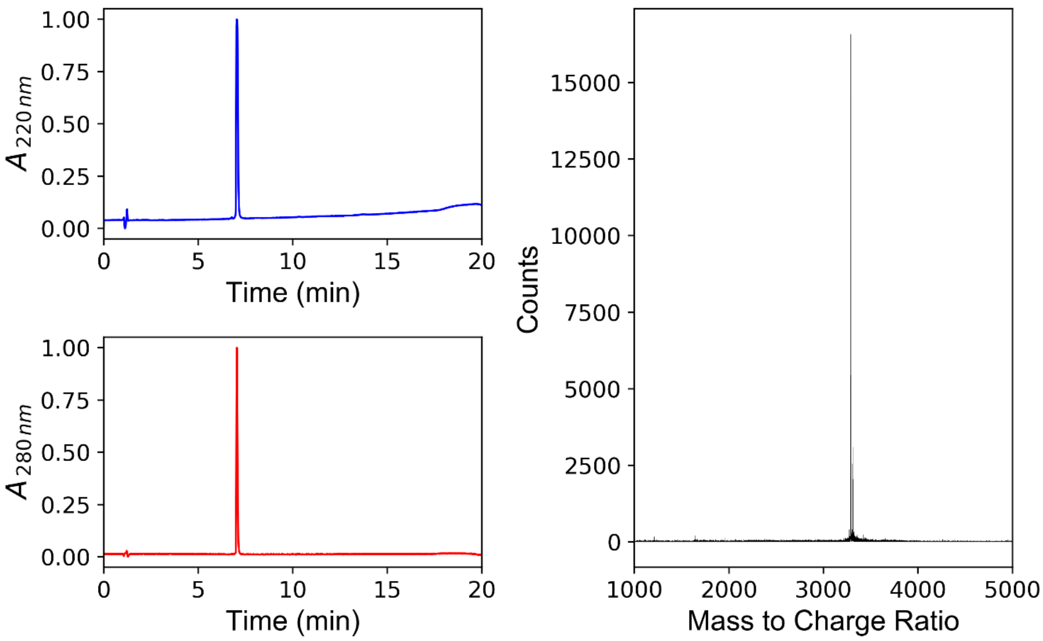

Figure S2j. Analytical HPLC and MS data for apCC-Tet-B-RRXS$_{20}$. (Left) Representative analytical HPLC chromatogram monitoring absorbance at 220 nm (blue) and 280 nm (red) of peptide apCC-Tet-B-RRXS$_{20}$. (Right) Representative mass spectrum of peptide apCC-Tet-B-RRXS$_{20}$ (black), with expected and observed masses of 3288.0 and 3286.0 Da, respectively.

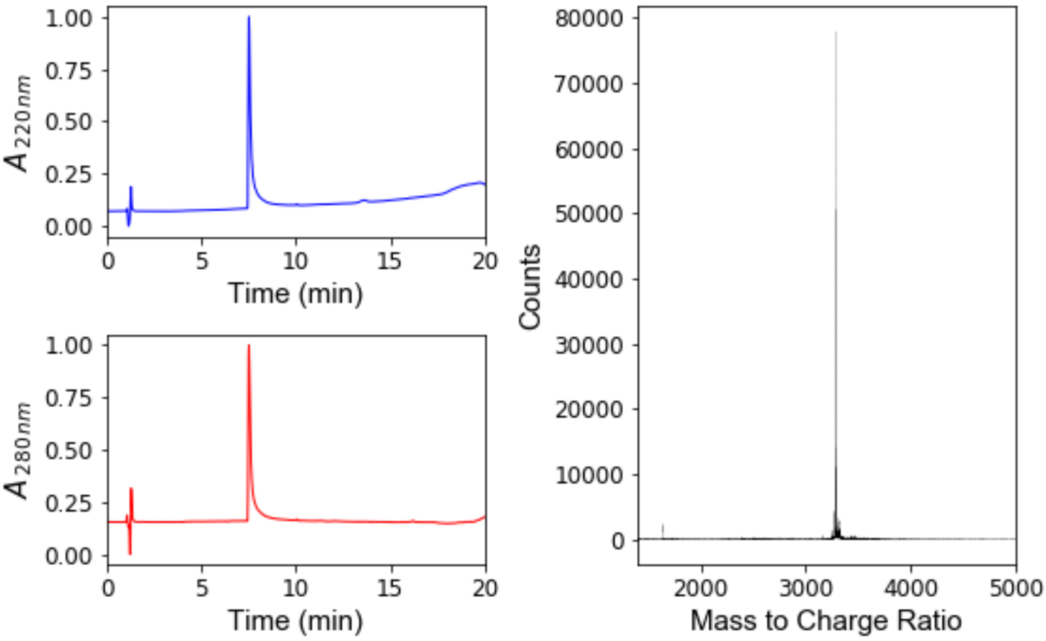

Figure S2k. Analytical HPLC and MS data for apCC-Tet-B-RRXS$_{13}$. (Left) Representative analytical HPLC chromatogram monitoring absorbance at 220 nm (blue) and 280 nm (red) of peptide apCC-Tet-B-RRXS$_{13}$. (Right) Representative mass spectrum of peptide apCC-Tet-B-RRXS$_{13}$ (black), with expected and observed masses of 3288.0 and 3289.4 Da, respectively.

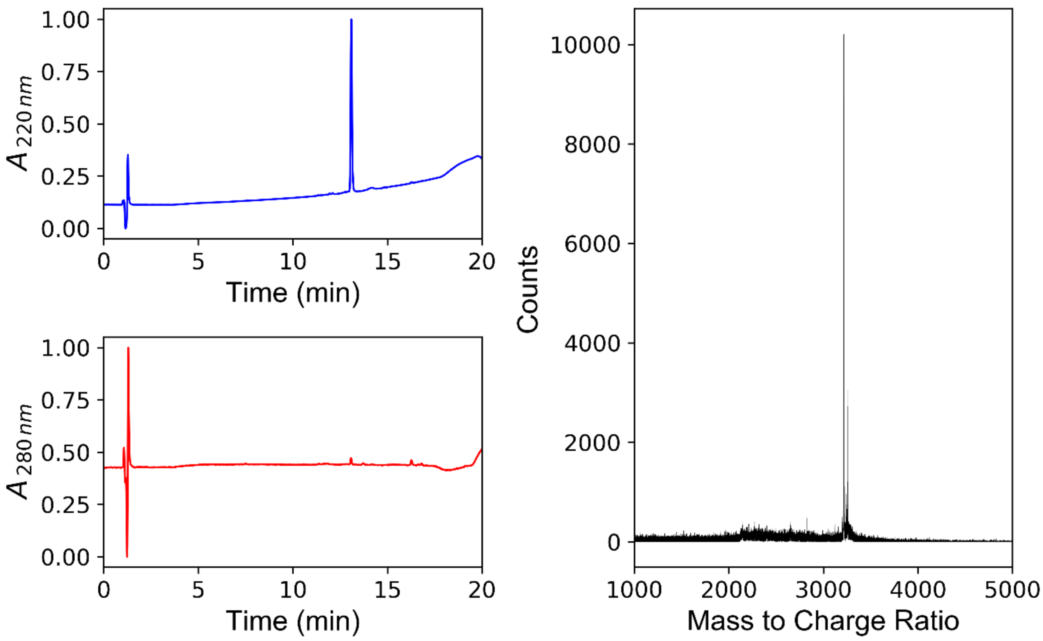

Figure S2l. Analytical HPLC and MS data for apCC-Tet-A-$S_{17}$$SeMet_4$. (Left) Representative analytical HPLC chromatogram monitoring absorbance at 220 nm (blue) and 280 nm (red) of peptide apCC-Tet-A-$S_{17}$$SeMet_4$. (Right) Representative mass spectrum of peptide apCC-Tet-A-S_{17} (black), with expected and observed masses of 3215.4 and 3217.1 Da, respectively.

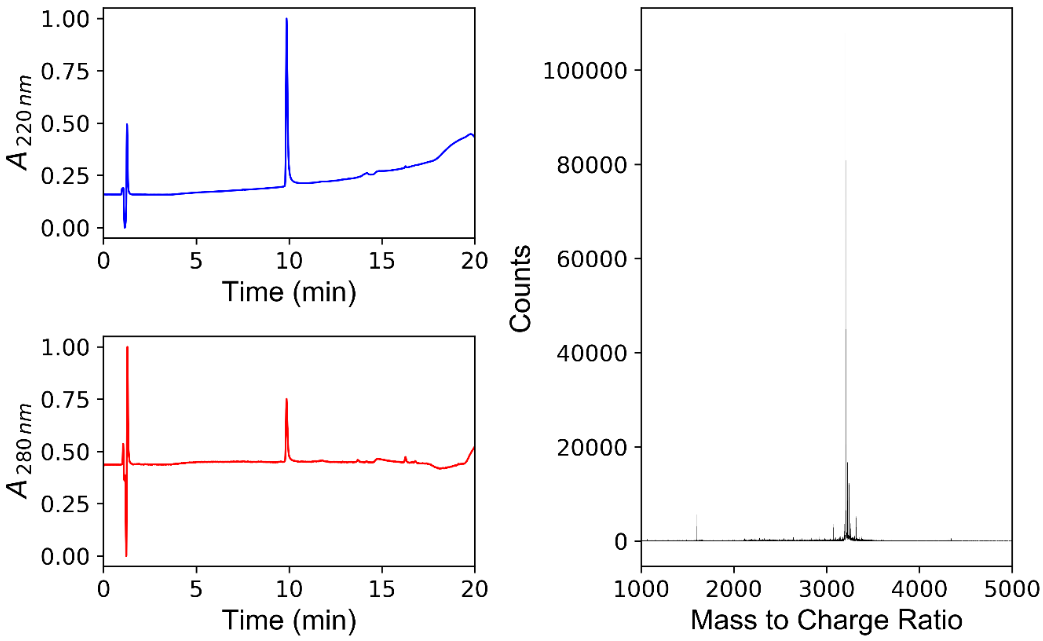

Figure S2m. Analytical HPLC and MS data for apCC-Tet-B-S_{20} $4CF_2$. (Left) Representative analytical HPLC chromatogram monitoring absorbance at 220 nm (blue) and 280 nm (red) of peptide apCC-Tet-B-S_{20} $4CF_2$. (Right) Representative mass spectrum of peptide apCC-Tet-B-S_{20} $4CF_2$ (black), with expected and observed masses of 3203.9 and 3204.2 Da, respectively.

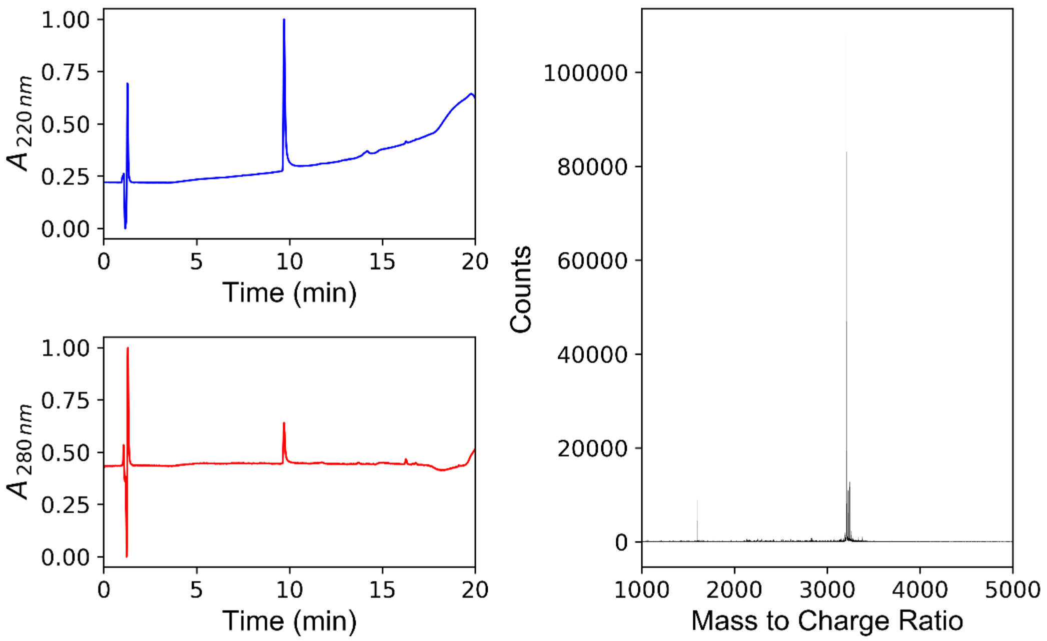

Figure S2n. Analytical HPLC and MS data for apCC-Tet-B-S_{20} $4CF_{25}$. (Left) Representative analytical HPLC chromatogram monitoring absorbance at 220 nm (blue) and 280 nm (red) of peptide apCC-Tet-B-S_{20} $4CF_{25}$. (Right) Representative mass spectrum of peptide apCC-Tet-B-S_{20} $4CF_{25}$ (black), with expected and observed masses of 3203.9 and 3203.4 Da, respectively.

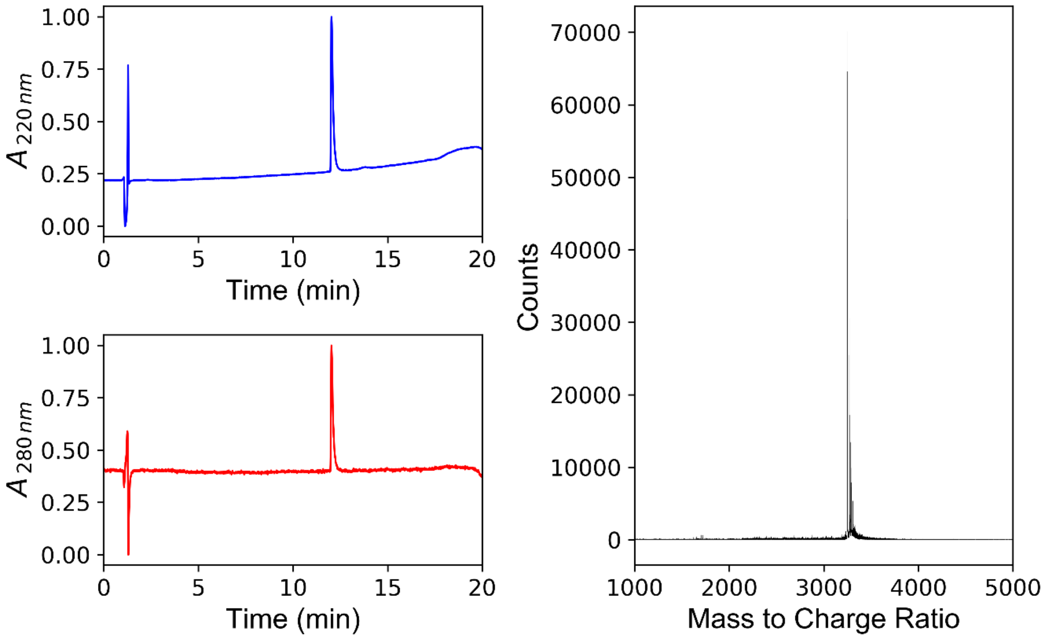

Figure S2o. Analytical HPLC and MS data for apCC-Tet. (Left) Representative analytical HPLC chromatogram monitoring absorbance at 220 nm (blue) and 280 nm (red) of peptide apCC-Tet. (Right) Representative mass spectrum of peptide apCC-Tet (black), with expected and observed masses of 3246.9 and 3247.6 Da, respectively.

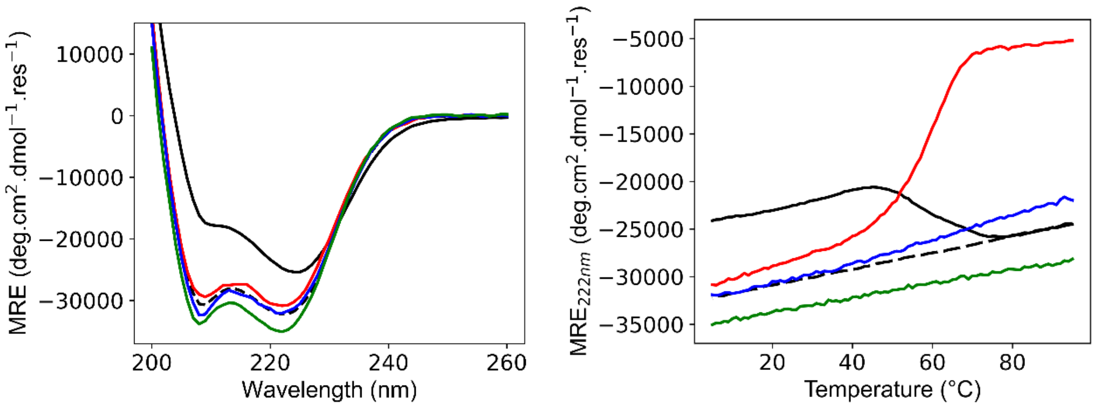

Figure S3. CD spectroscopy data for initial antiparallel hetero-tetramer peptide designs. (Left) Representative CD spectra at 5 °C, and (right) variable temperature CD measurements monitoring mean residue ellipticity at 222 nm (MRE_{222}) between 5 and 95 °C. Key: apCC-Tet-A1 (red); apCC-Tet-B1 (blue); apCC-Tet (green); apCC-Tet-A1 plus apCC-Tet-B1 (black, with after heating scan and refolding measurements dashed). Measurements were made in PBS (pH 7.4) at 10 μM individual peptide.

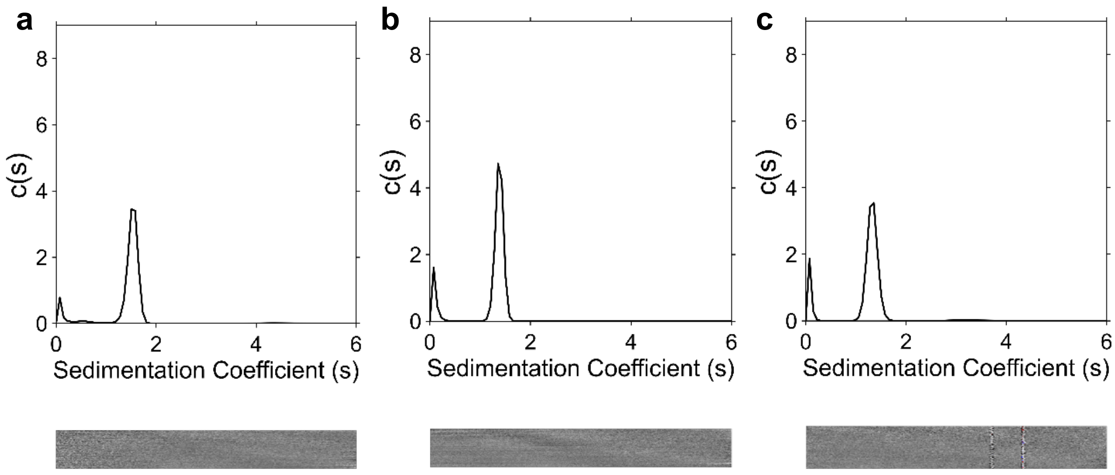

Figure S4. Sedimentation velocity AUC data for initial antiparallel hetero-tetramer designs. SV c(s) distribution (top) and residuals (bottom) for; (a) apCC-Tet-A1 returning molecular weight of 14511 Da (4.5 x monomer mass). (b) apCC-Tet-B1 returning molecular weight of 16770 Da (5.2 x monomer mass). (c) apCC-Tet-A1 plus apCC-Tet-B1 (annealed) returning molecular weight of 13774 Da (4.2 x monomer mass). Measurements were made in PBS (pH 7.4).

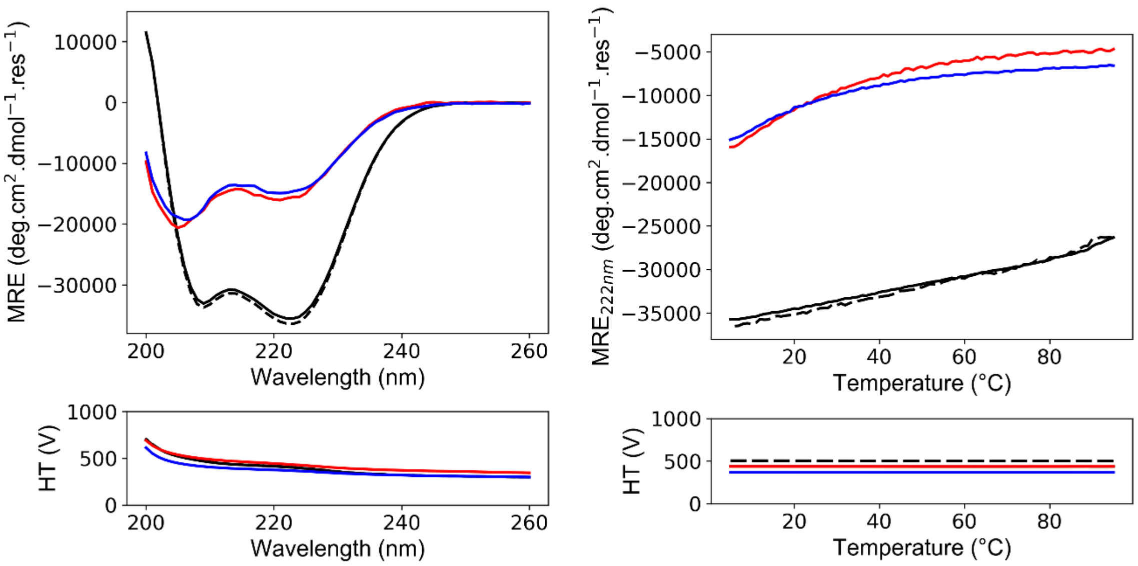

Figure S5. CD spectroscopy data for antiparallel heterotetramer apCC-Tet-A$_2$B$_2$. (Left) 5 °C CD spectra data (top) and HT traces (bottom) for the peptides apCC-Tet-A-S$_{17}$ (red), apCC-Tet-B-S$_{20}$ (blue), apCC-Tet-A-S$_{17}$ plus apCC-Tet-B-S$_{20}$ (black) and apCC-Tet-A-S$_{17}$ plus apCC-Tet-B-S$_{20}$ after heating (black dash). (Right) Variable temperature (5 – 95 – 5 °C) CD measurement (top) and HT traces (bottom) for the peptides apCC-Tet-A-S$_{17}$ (red), apCC-Tet-B-S$_{20}$ (blue), apCC-Tet-A-S$_{17}$ plus apCC-Tet-B-S$_{20}$ (black)(all 5 – 95 °C) and apCC-Tet-A-S$_{17}$ plus apCC-Tet-B-S$_{20}$ (black dash, 95-5 °C). Measurements were taken with 10 μM individual peptide in PBS (pH 7.4).

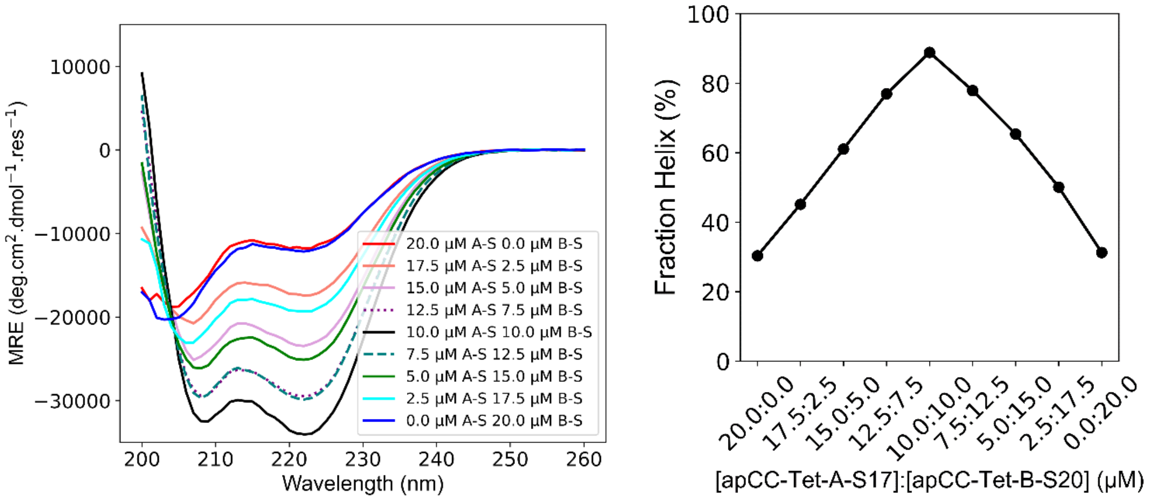

Figure S6. Continuous variation data for heterotetramer apCC-Tet-A-S$_2$B-S$_2$. (Left) CD spectra at different ratios of apCC-Tet-A-S$_{17}$ and apCC-Tet-B-S$_{20}$. Total peptide concentration was maintained at 20 μM and all spectra measured at 20 °C in PBS (pH 7.4). (Right) Job plot displaying fraction helix for each of the different stoichiometric ratios of apCC-Tet-A-S$_{17}$ and apCC-Tet-B-S$_{20}$.

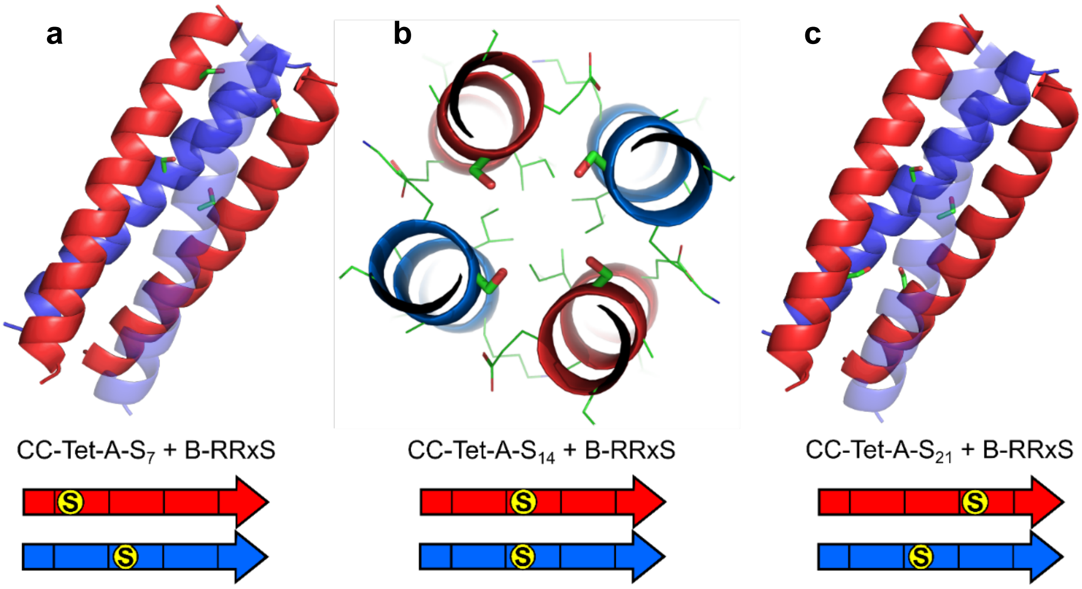

Figure S7. Modelling the location of serine in parallel heterotetramer designs. (a) CC-Tet-A-S_7 & CC-Tet-B-RRXS$_{14}$, (b) CC-Tet-A-S_{14} & CC-Tet-B-RRXS$_{14}$ and (c) CC-Tet-A-S_{21} & CC-Tet-B-RRXS$_{14}$, heterotetramer models with cartoon below to show the location of serine-containing layer (front helix is partially transparent in a and c to allow a better view of the core). Serine residues are shown as green sticks. Models were produced and optimized in ISAMBARD[1] based on X-ray crystal structures for CC-Tet (PDB code 3R4A)[2].

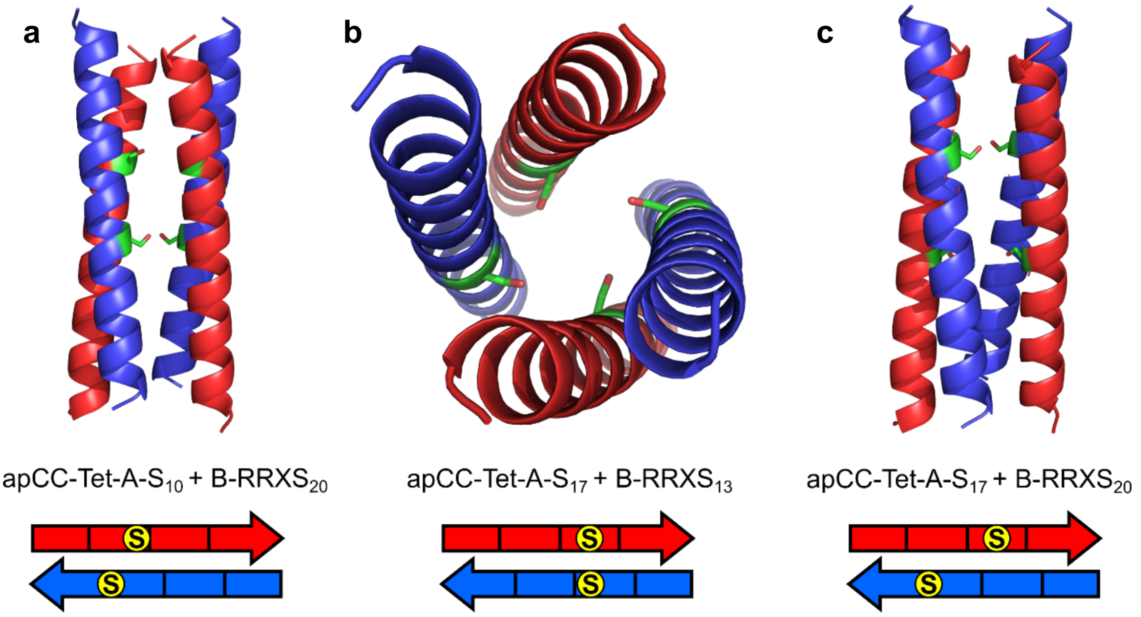

Figure S8. Modelling the location of serine in antiparallel heterotetramer designs. (a) apCC-Tet-A-S_{10} plus apCC-Tet-B-RRXS$_{20}$, (b) apCC-Tet-A-S_{17} plus apCC-Tet-B-RRXS$_{13}$ and (c) apCC-Tet-A-S_{17} plus apCC-Tet-B-RRXS$_{20}$, heterotetramer complex models with cartoon below to show serine-containing layer. Serine residues are shown as green sticks. Models were produced in PyMol[3] based on X-ray crystal structures for apCC-Tet (PDB code 6Q5S)[4].

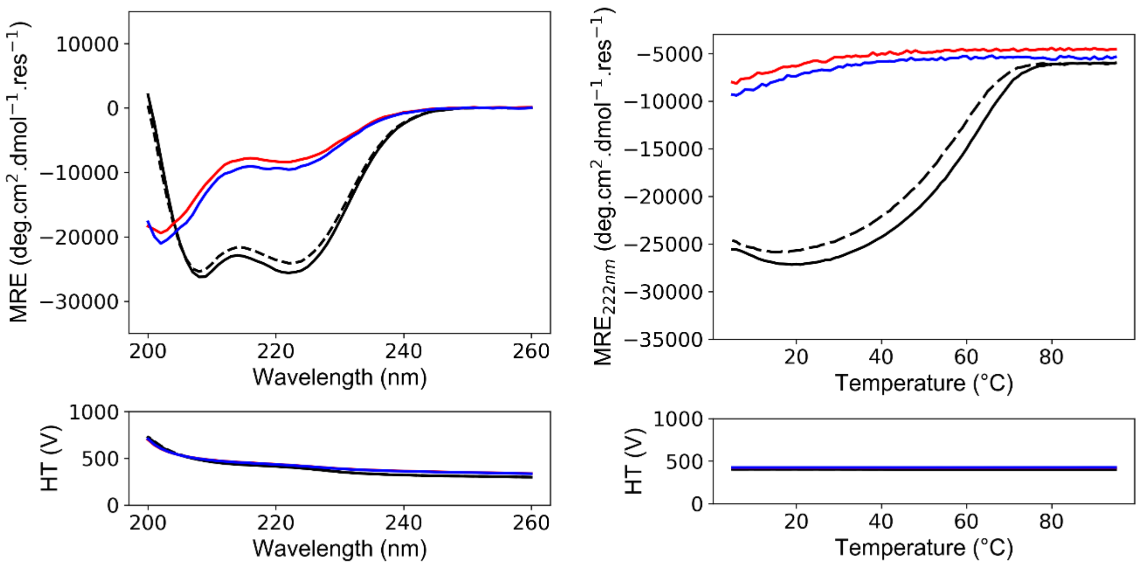

Figure S9. CD spectroscopy data for CC-Tet-A-S$_{14}$ and CC-Tet-B-RRXS$_{14}$. (Left, top) 5 °C CD spectra data and (left, bottom) HT traces for the peptides CC-Tet-A-S$_{14}$ (red), CC-Tet-B-RRXS$_{14}$ blue), CC-Tet-A-S$_{14}$ plus CC-Tet-B-RRXS$_{14}$ (black) and CC-Tet-A-S$_{14}$ plus CC-Tet-B-RRXS$_{14}$ after heating (black dash). (Right, top) Variable temperature (5 – 95 – 5 °C) CD measurement and (right, bottom) HT traces for the peptides CC-Tet-A-S$_{14}$ (red), CC-Tet-B-RRXS$_{14}$ (blue), CC-Tet-A-S$_{14}$ plus CC-Tet-B-RRXS$_{14}$ (black) (all 5 – 95 °C) and CC-Tet-A-S$_{14}$ plus CC-Tet-B-RRXS$_{14}$ (black dash, 95–5 °C). Measurements recorded with 10 μM individual peptide in PBS (pH 7.4).

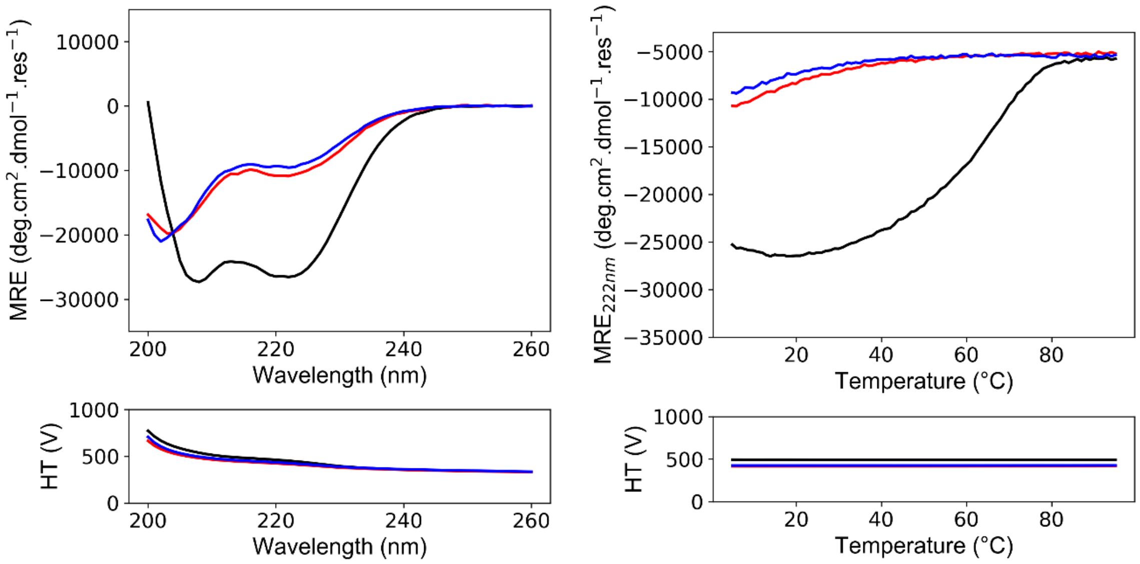

Figure S10. CD spectroscopy data for CC-Tet-A-S$_7$ and CC-Tet-B-RRXS$_{14}$. (Left) 5 °C CD spectra data (top) and HT traces (bottom) for the peptides CC-Tet-A-S$_7$ (red), CC-Tet-B-RRXS$_{14}$ (blue) and CC-Tet-A-S$_7$ plus CC-Tet-B-RRXS$_{14}$ (black). (Right) Variable temperature (5 – 95 °C) CD measurement (top) and HT traces (bottom) for the peptides CC-Tet-A-S$_7$ (red), CC-Tet-B-RRXS$_{14}$ (blue) and CC-Tet-A-S$_7$ plus CC-Tet-B-RRXS$_{14}$ (black). Measurements recorded with 10 μM individual peptide in PBS (pH 7.4).

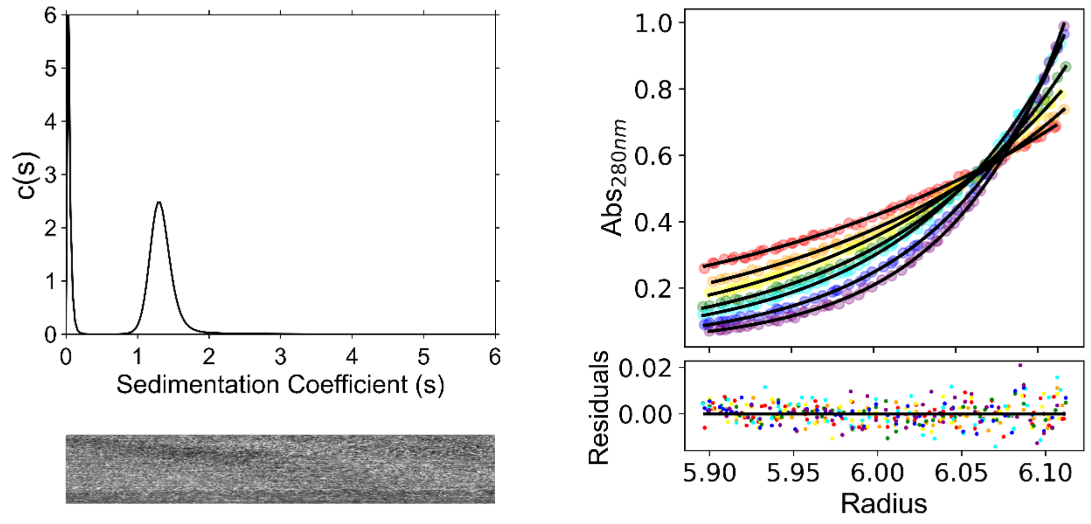

Figure S11. Analytical ultracentrifugation data for CC-Tet-A-S$_7$ and CC-Tet-B-RRXS$_{14}$. (Left) SV and (right) SE data for hetero-tetramer CC-Tet-A-S$_7$ and CC-Tet-B-RRXS$_{14}$ returning molecular weights of 12801 Da (3.9 x monomer mass) and 13003 Da (4.0 x monomer mass, 95 % confidence limits 12937–13079 Da), respectively. For SV data; c(s) distribution (top) and residuals (bottom) and for SE fits (top) and residuals (bottom). All measurements were made in PBS (pH 7.4).

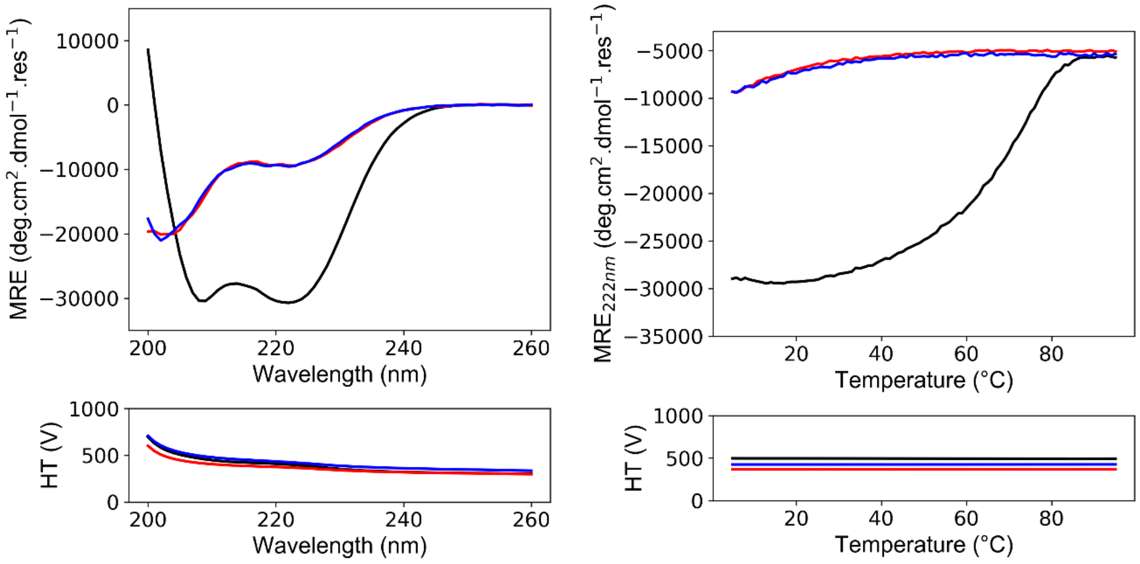

Figure S12. CD spectroscopy data for CC-Tet-A-S$_{21}$ and CC-Tet-B-RRXS$_{14}$. (Left) 5 °C CD spectra data (top) and HT traces (bottom) for the peptides CC-Tet-A-S$_{21}$ (red), CC-Tet-B-RRXS$_{14}$ (blue) and CC-Tet-A-S$_{21}$ plus CC-Tet-B-RRXS$_{14}$ (black). (Right) Variable temperature (5–95 °C) CD measurement (top) and HT traces (bottom) for the peptides CC-Tet-A-S$_{21}$ (red), CC-Tet-B-RRXS$_{14}$ (blue) and CC-Tet-A-S$_{21}$ plus CC-Tet-B-RRXS$_{14}$ (black). Measurements were made with 10 μM individual peptide in PBS (pH 7.4).

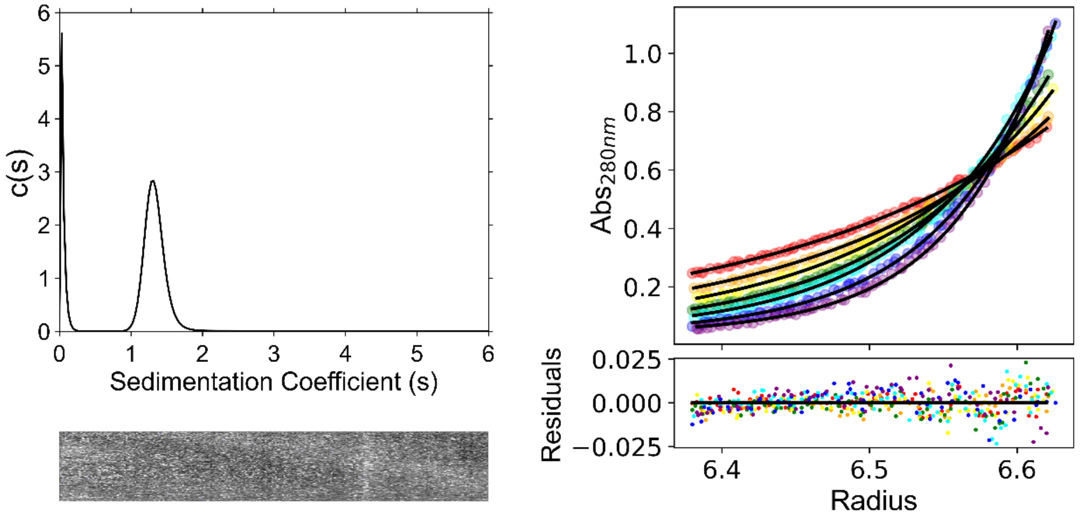

Figure S13. Analytical ultracentrifugation data for CC-Tet-A-S$_{21}$ and CC-Tet-B-RRXS$_{14}$. (Left) SV and (right) SE data for hetero-tetramer CC-Tet-A-S$_{21}$ and CC-Tet-B-RRXS$_{14}$ returning molecular weights of 13802 Da (4.2 x monomer mass) and 12871 Da (4.0 x monomer mass, 95 % confidence limits 12804 – 12933 Da) respectively. For SV data; c(s) distribution (top) and residuals (bottom) and for SE fits (top) and residuals (bottom). All measurements were recorded in PBS (pH 7.4).

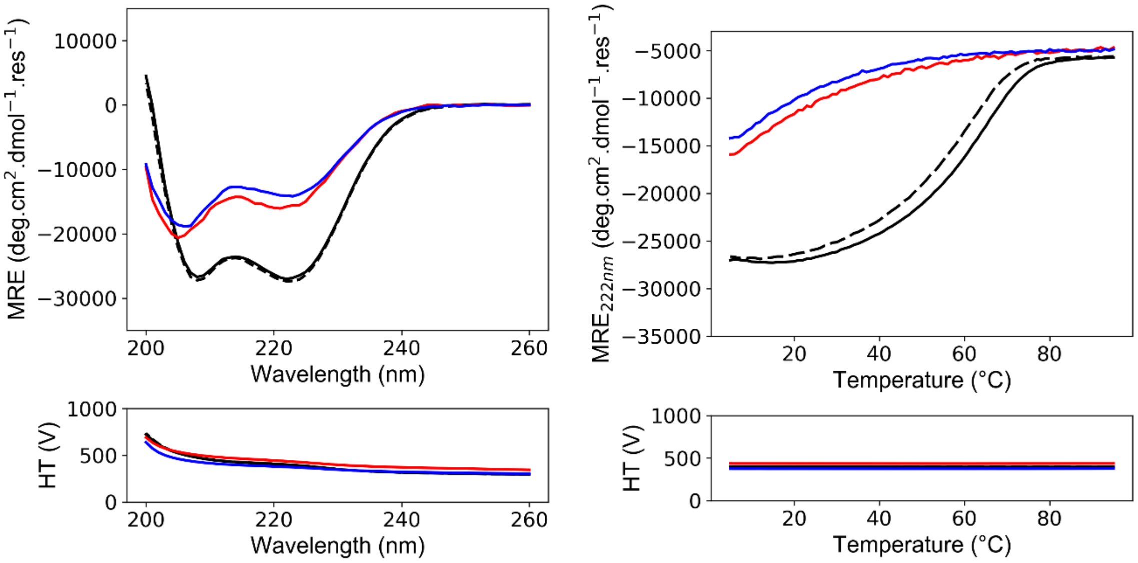

Figure S14. CD spectroscopy data for apCC-Tet-A-S$_{17}$ and apCC-Tet-B-RRXS$_{20}$. (Left) 5 °C CD spectra data (top) and HT traces (bottom) for the peptides apCC-Tet-A-S$_{17}$ (red), apCC-Tet-B-RRXS$_{20}$ (blue), apCC-Tet-A-S$_{17}$ plus apCC-Tet-B-RRXS$_{20}$ (black) and apCC-Tet-A-S$_{17}$ plus apCC-Tet-B-RRXS$_{20}$ after heating (black dash). (Right) Variable temperature (5 – 95 °C) CD measurement (top) and HT traces (bottom) for the peptides apCC-Tet-A-S$_{17}$ (red), apCC-Tet-B-RRXS$_{20}$ (blue), apCC-Tet-A-S$_{17}$ plus apCC-Tet-B-RRXS$_{20}$ (black) and apCC-Tet-A-S$_{17}$ plus apCC-Tet-B-RRXS$_{20}$ (95 – 5 °C cool, black dash). Measurements recorded with 10 μM individual peptide in PBS (pH 7.4).

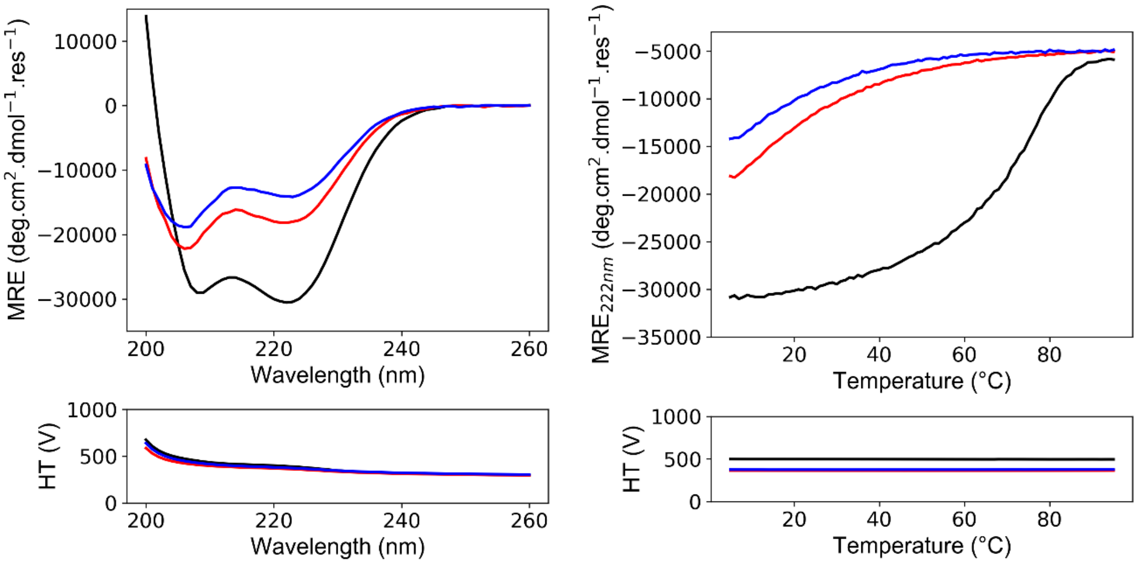

Figure S15. CD spectroscopy data for apCC-Tet-A-S$_{10}$ and apCC-Tet-B-RRXS$_{20}$. (Left) 5 °C CD spectra data (top) and HT traces (bottom) for the peptides apCC-Tet-A-S$_{10}$ (red), apCC-Tet-B-RRXS$_{20}$ (blue) and apCC-Tet-A-S$_{10}$ plus apCC-Tet-B-RRXS$_{20}$ (black). (Right) Variable temperature (5 – 95 °C) CD measurement (top) and HT traces (bottom) for the peptides apCC-Tet-A-S$_{10}$ (red), apCC-Tet-B-RRXS$_{20}$ (blue) and apCC-Tet-A-S$_{10}$ plus apCC-Tet-B-RRXS$_{20}$ (black). Measurements made with 10 μM individual peptide in PBS (pH 7.4).

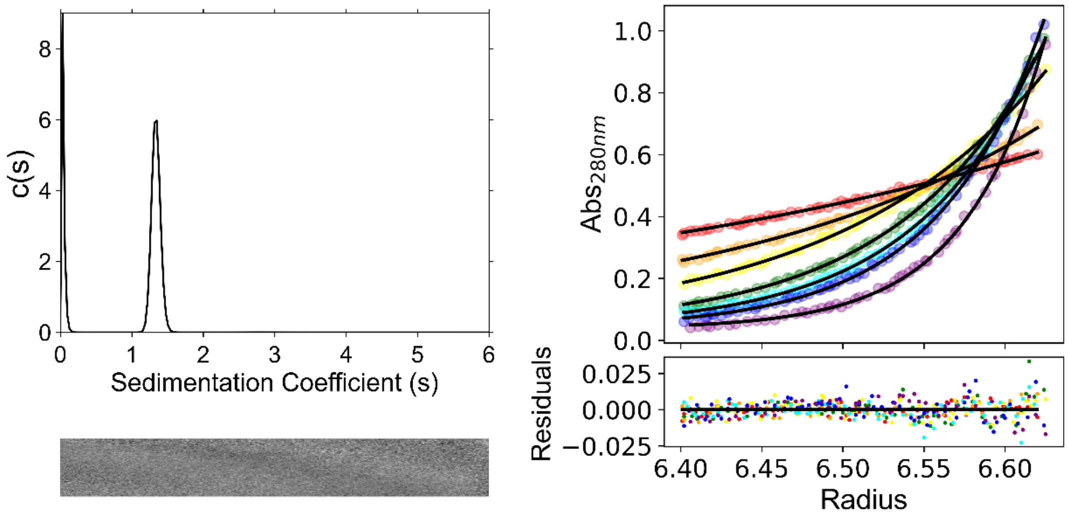

Figure S16. Analytical ultracentrifugation data for apCC-Tet-A-S$_{10}$ and apCC-Tet-B-RRXS$_{20}$. (Left) SV and (right) SE data for hetero-tetramer apCC-Tet-A-S$_{10}$ and apCC-Tet-RRXS$_{20}$ returning molecular weights of 13064 Da (4.0 x monomer mass) and 12216 Da (3.8 x monomer mass, 95 % confidence limits 12139 – 12293 Da) respectively. For SV data; c(s) distribution (top) and residuals (bottom) and for SE fits (top) and residuals (bottom). All measurements were recorded in PBS (pH 7.4).

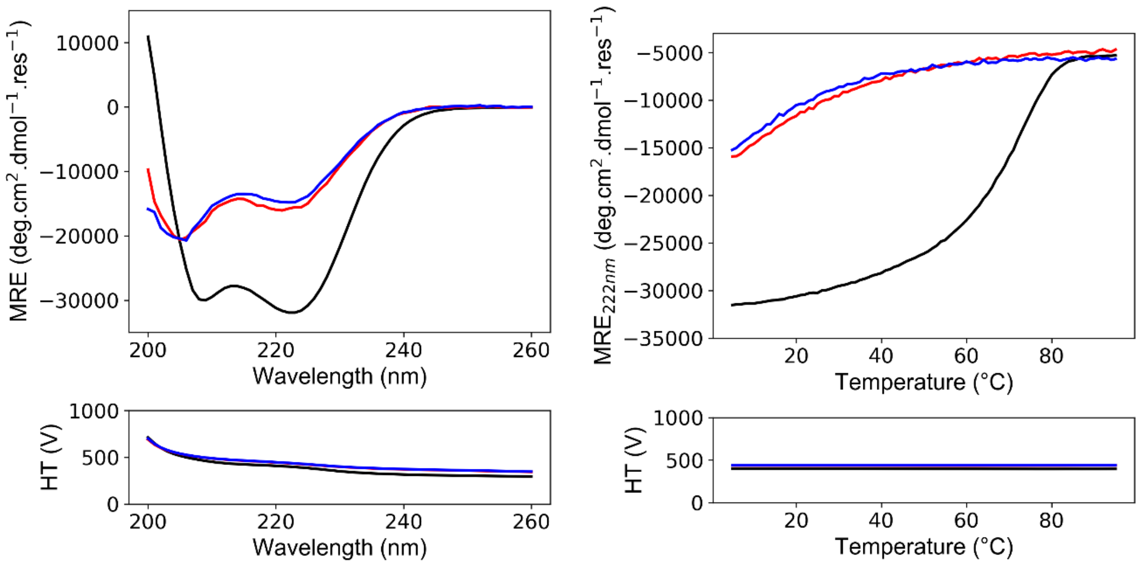

Figure S17. CD spectroscopy data for apCC-Tet-A-S_{17} and apCC-Tet-B-RRXS_{13}. (Left) 5 °C CD spectra data (top) and HT traces (bottom) for the peptides apCC-Tet-A-S_{17} (red), apCC-Tet-B-RRXS_{13} (blue) and apCC-Tet-A-S_{17} plus apCC-Tet-B-RRXS_{13} (black). (Right) Variable temperature (5 – 95 °C) CD measurement (top) and HT traces (bottom) for the peptides apCC-Tet-A-S_{17} (red), apCC-Tet-B-RRXS_{13} (blue) and apCC-Tet-A-S_{17} plus apCC-Tet-B-RRXS_{13} (black). Measurements recorded with 10 μM individual peptide in PBS (pH 7.4).

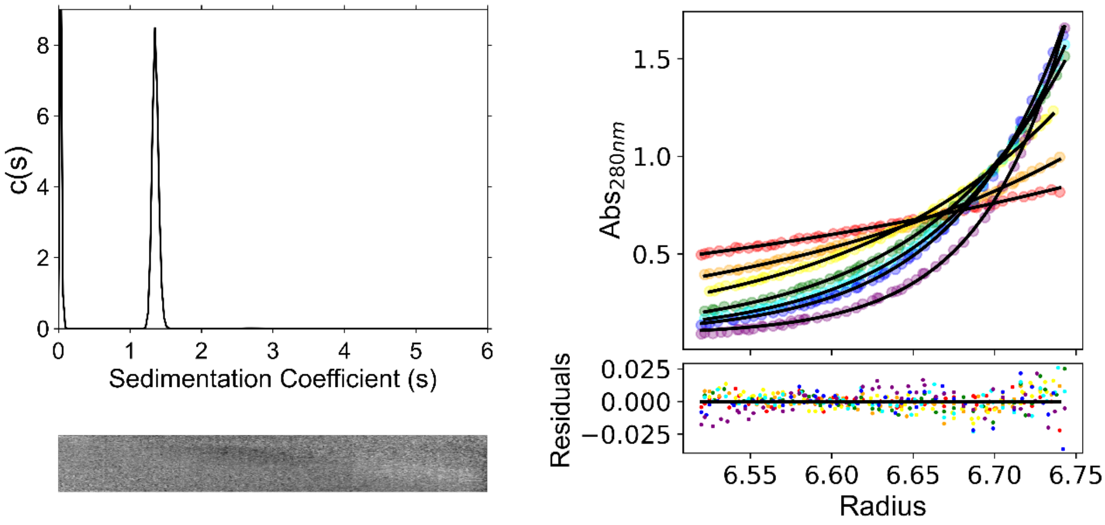

Figure S18. Analytical ultracentrifugation data for apCC-Tet-A-S_{17} and apCC-Tet-B-RRXS_{13}. (Left) SV and (right) SE data for hetero-tetramer apCC-Tet-A-S_{17} and apCC-Tet-B-RRXS_{13} returning molecular weights of 13254 Da (4.1 x monomer mass) and 11591 Da (3.6 x monomer mass, 95 % confidence limits 11524 – 11654 Da) respectively. For SV data; c(s) distribution (top) and residuals (bottom) and for SE fits (top) and residuals (bottom). All measurements were recorded in PBS (pH 7.4).

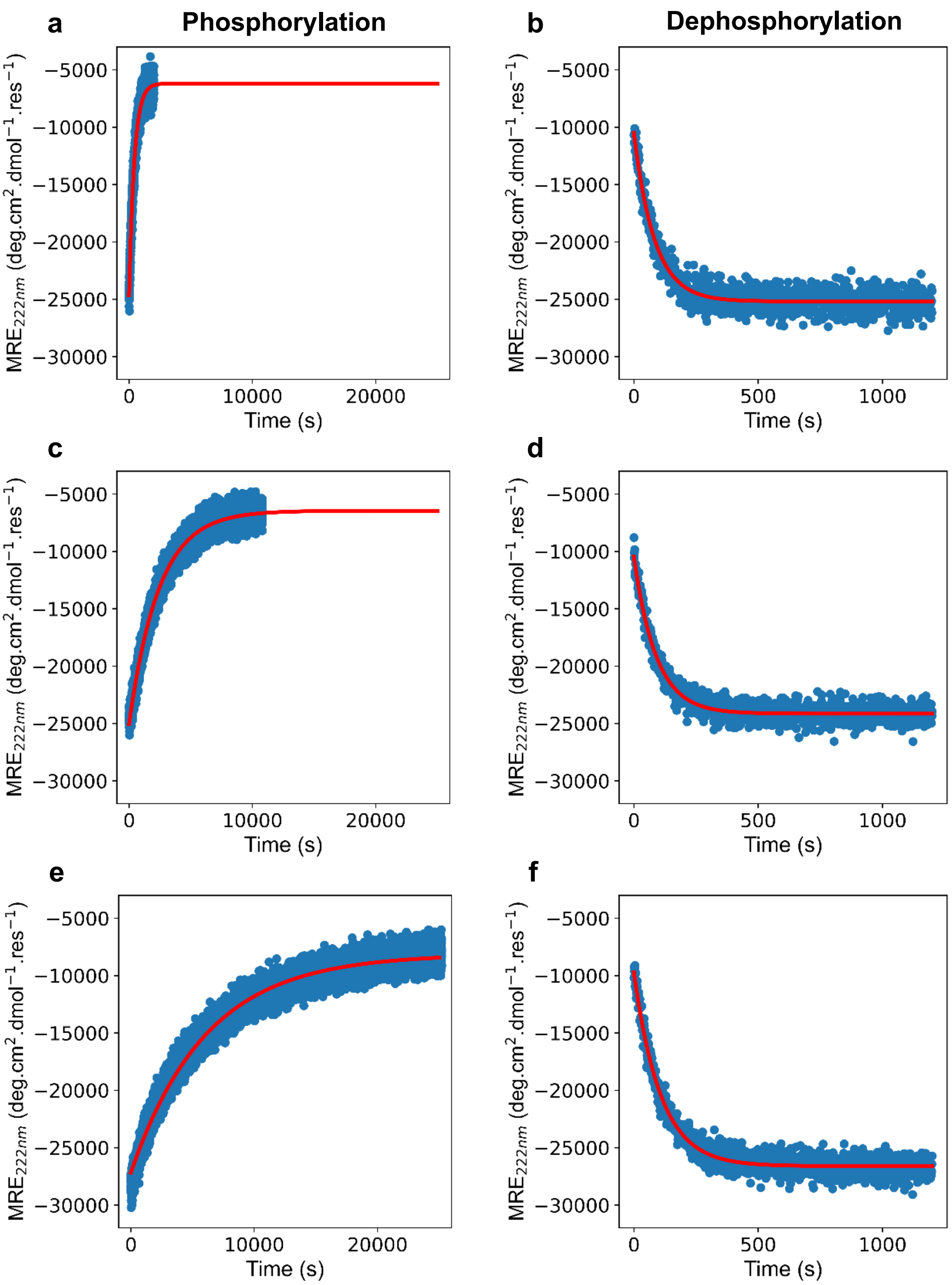

Figure S19. Phosphorylation and dephosphorylation of CC-Tet-B-RRXS$_{14}$ in combination with CC-Tet-A-S$_{14}$, CC-Tet-A-S$_7$ or CC-Tet-A-S$_{21}$ monitored by CD spectroscopy. (a) Phosphorylation and (b) dephosphorylation, of CC-Tet-A-S$_{14}$ plus CC-Tet-B-RRXS$_{14}$. (c) Phosphorylation and (d) dephosphorylation, of CC-Tet-A-S$_7$ plus CC-Tet-B-RRXS$_{14}$. (e) Phosphorylation and (f) dephosphorylation, of CC-Tet-A-S$_{21}$ plus CC-Tet-B-RRXS$_{14}$. For all a representative reaction is shown in blue, and an exponential fit shown in red. PKA concentration 5 U/μL, with phosphorylation reaction carried out in phosphorylation buffer. LPP concentration 2 U/μL, with dephosphorylation reaction carried out in phosphorylation buffer plus $MnCl_2$. Phosphorylation and dephosphorylation reactions were carried out with 10 and 9.85 μM of each peptide respectively.

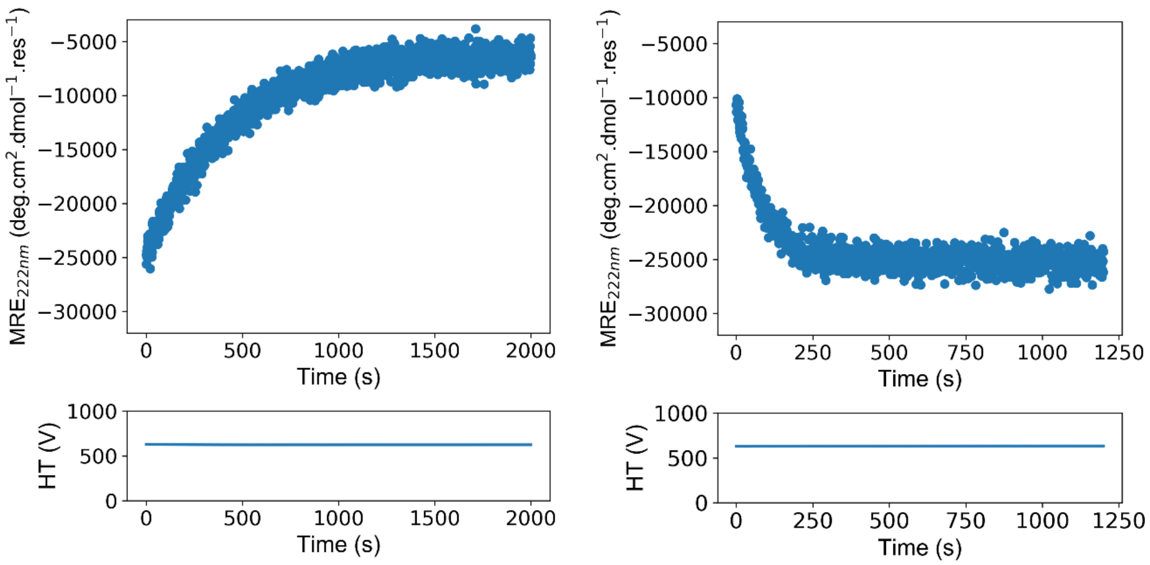

Figure S20. Representative phosphorylation and dephosphorylation reaction for CC-Tet-A-S$_{14}$ plus CC-Tet-B-RRXS$_{14}$. Phosphorylation (left) and dephosphorylation (right) at 10 µM individual peptide, with mean residue ellipticity at 222 nm (MRE$_{222}$) (top), and HT traces (bottom). PKA concentration 5 U/µL, with phosphorylation reaction carried out in phosphorylation buffer. LPP concentration 2 U/µL, with dephosphorylation reaction carried out in phosphorylation buffer plus $MnCl_2$.

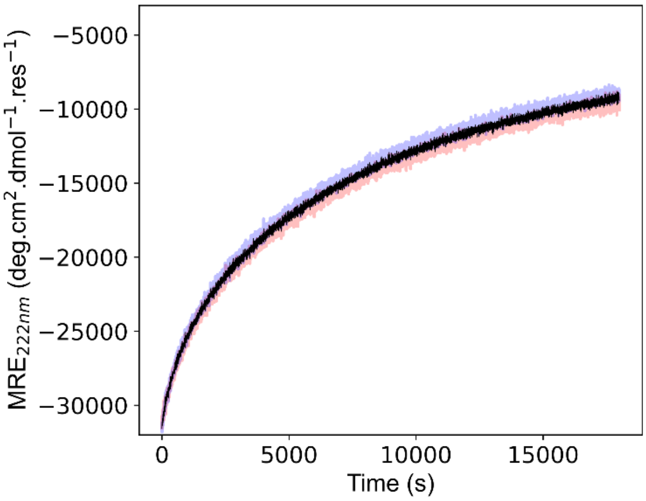

Figure S21. Monitoring phosphorylation reaction of 50 µM CC-Tet-A-S$_{14}$ and 50 µM CC-Tet-B-RRXS$_{14}$ by CD spectroscopy. Phosphorylation of CC-Tet-A-S$_{14}$ and CC-Tet-B-RRXS$_{14}$ hetero-tetramer monitored by mean residue ellipticity at 222 nm (MRE$_{222}$), using 5 U/µL PKA and 100 µM total peptide concentration (n = 2) individual peptide. Color represents individual replicates and black is the mean. Reactions carried out in phosphorylation buffer.

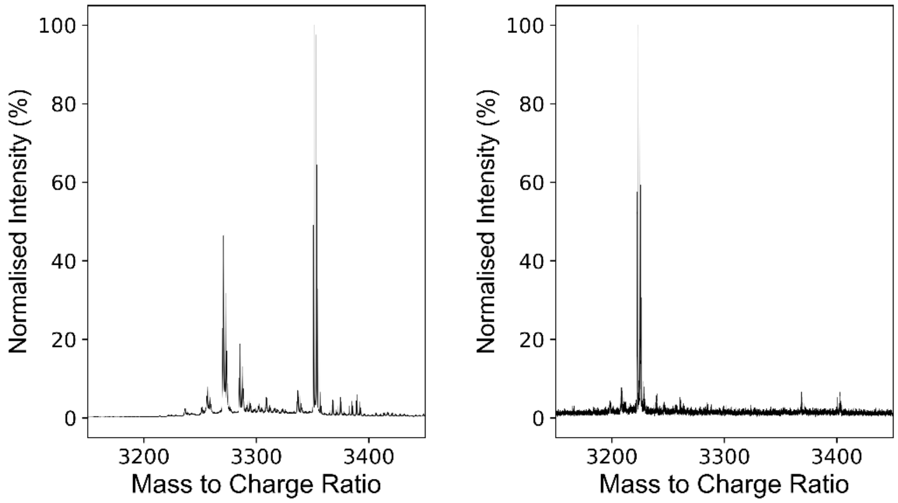

Figure S22. Phosphorylation of the parallel basic peptide confirmed by MS analysis. (Left) Representative mass spectrum for CC-Tet-B-RRXS$_{14}$ after incubation with PKA, with expected and observed masses of 3272.1 and 3351.8 Da respectively. The mass is 79.7 Da larger than the peptide on its own due to the addition of a phosphoryl group. (Right) Representative mass spectrum for CC-Tet-A-S$_{14}$ after incubation with PKA, with expected and observed masses of 3223.7 and 3223.5 Da respectively. PKA concentration 5 U/μL, with reaction carried out in phosphorylation buffer.

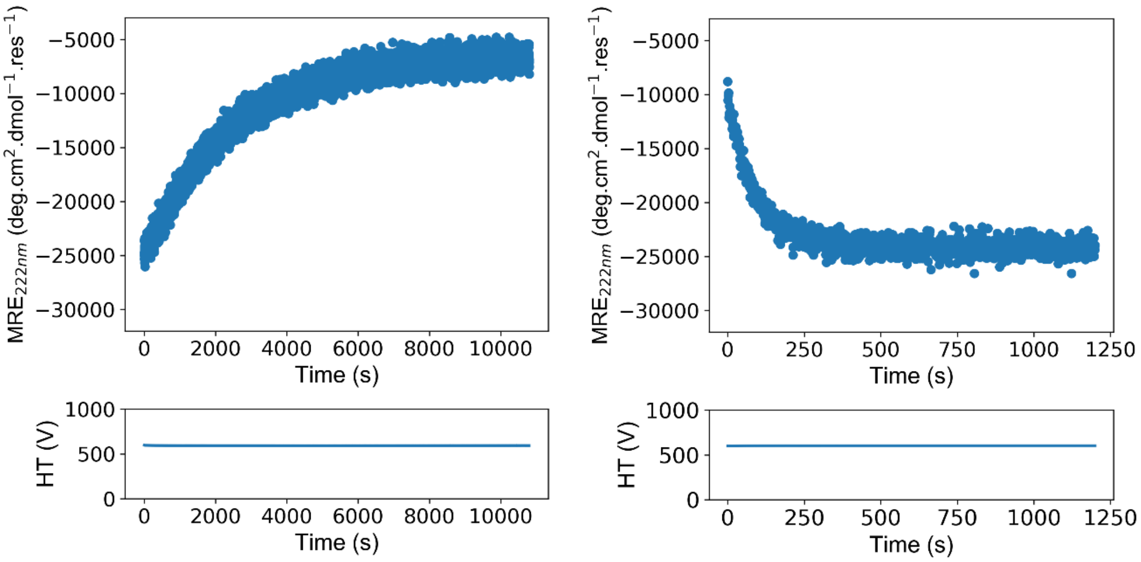

Figure S23. Representative phosphorylation and dephosphorylation reaction for CC-Tet-A-S$_7$ plus CC-Tet-B-RRXS$_{14}$. Phosphorylation (left) and dephosphorylation (right) at 10 and 9.85 μM individual peptide respectively, with mean residue ellipticity at 222 nm (MRE$_{222}$) (top), and HT traces (bottom). PKA concentration 5 U/μL, with phosphorylation reaction carried out in phosphorylation buffer. LPP concentration 2 U/μL, with dephosphorylation reaction carried out in phosphorylation buffer plus $MnCl_2$.

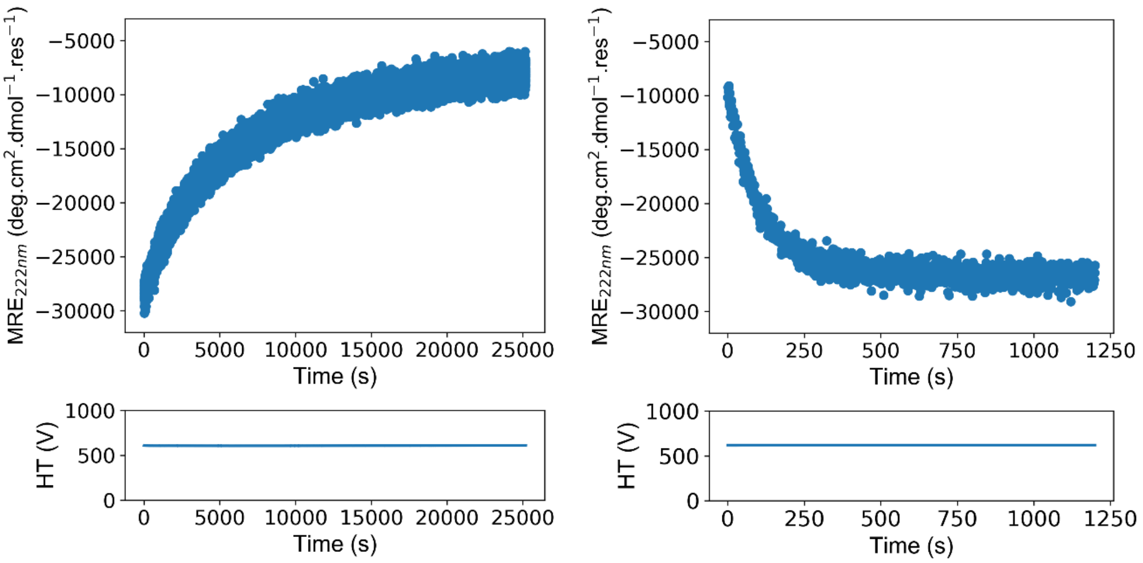

Figure S24. Representative phosphorylation and dephosphorylation reaction for CC-Tet-A-S$_{21}$ plus CC-Tet-B-RRXS$_{14}$. Phosphorylation (left) and dephosphorylation (right) at 10 and 9.85 μM individual peptide respectively, with mean residue ellipticity at 222 nm (MRE$_{222}$) (top), and HT traces (bottom). PKA concentration 5 U/μL, with phosphorylation reaction carried out in phosphorylation buffer. LPP concentration 2 U/μL, with dephosphorylation reaction carried out in phosphorylation buffer plus $MnCl_2$.

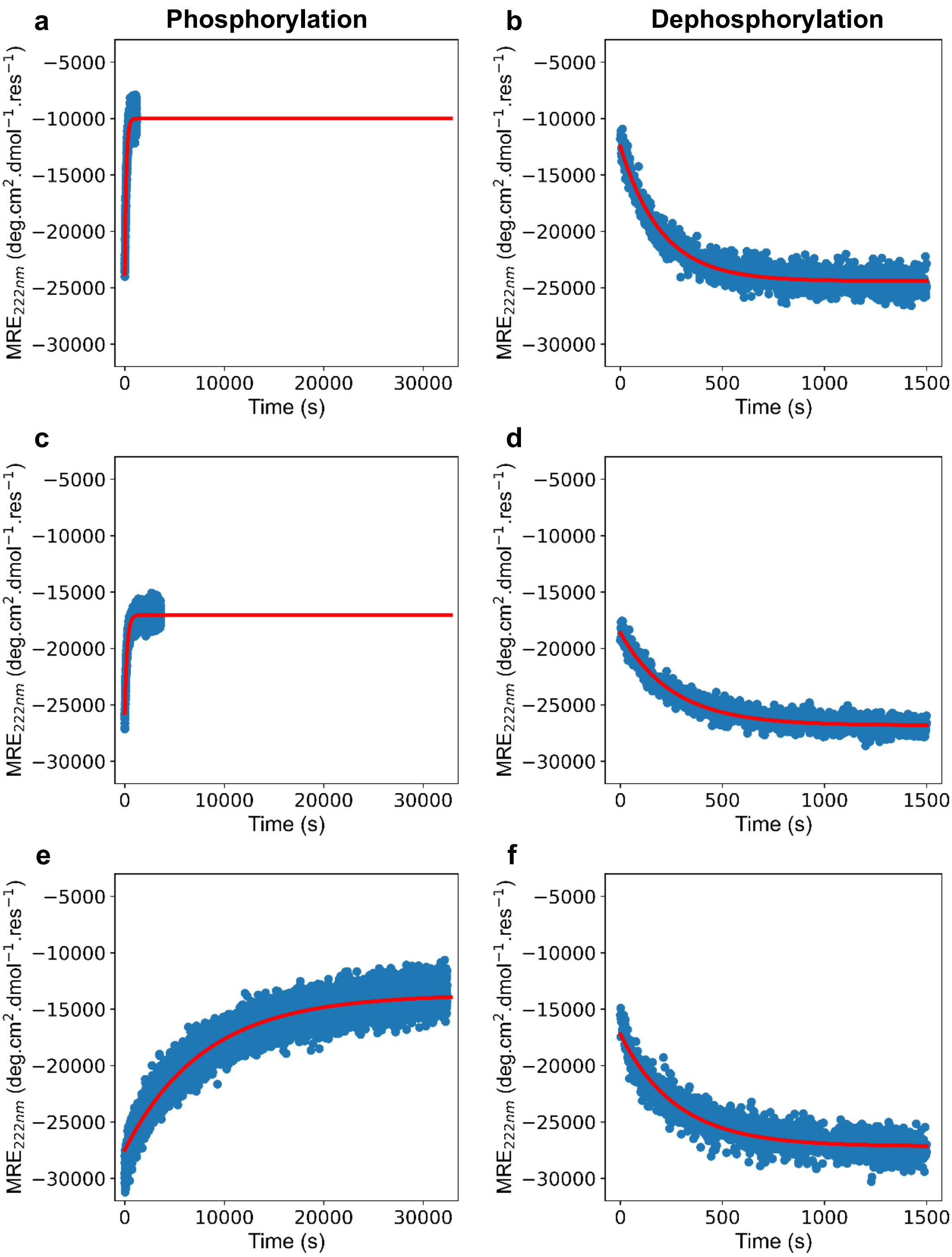

Figure S25. Phosphorylation and dephosphorylation of apCC-Tet-A-S17, apCC-Tet-A-S10, apCC-Tet-B-RRXS20 and apCC-Tet-B-RRXS13 monitored by CD. (a) Phosphorylation and (b) dephosphorylation, of apCC-Tet-A-S17 plus apCC-Tet-B-RRXS20. (c) Phosphorylation and (d) dephosphorylation, of apCC-Tet-A-S10 plus apCC-Tet-B-RRXS20. (e) Phosphorylation and (f) dephosphorylation, of apCC-Tet-A-S17 plus apCC-Tet-B-RRXS13. For all a representative reaction is shown in blue, and an exponential fit shown in red. PKA concentration 1 U/μL for apCC-Tet-A-S17 plus apCC-Tet-B-RRXS20, and 5 U/μL for others, with phosphorylation reaction carried out in phosphorylation buffer. LPP concentration 2 U/μL, with dephosphorylation reaction carried out in phosphorylation buffer plus $MnCl_2$ for all. Phosphorylation and dephosphorylation reactions were carried out with 10 and 9.85 μM of each peptide respectively.

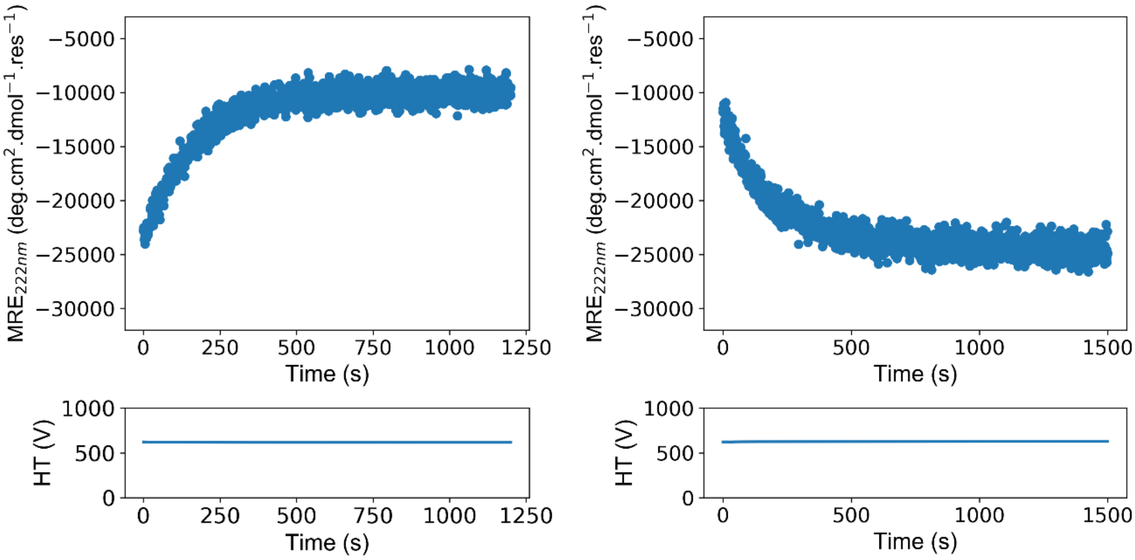

Figure S26. Representative phosphorylation and dephosphorylation reaction for apCC-Tet-A-S$_{17}$ plus apCC-Tet-B-RRXS$_{20}$. Phosphorylation (left) and dephosphorylation (right) at 10 and 9.85 μM individual peptide respectively, with mean residue ellipticity at 222 nm (MRE$_{222}$) (top), and HT traces (bottom). PKA concentration 1 U/μL, with phosphorylation reaction carried out in phosphorylation buffer. LPP concentration 2 U/μL, with dephosphorylation reaction carried out in phosphorylation buffer plus $MnCl_2$.

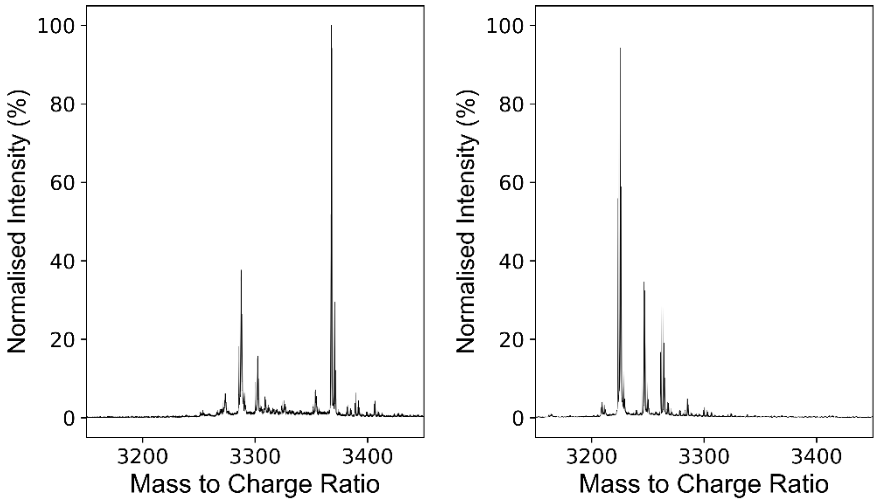

Figure S27. Phosphorylation of the antiparallel basic peptide confirmed by MS analysis. (Left) Representative mass spectrum for apCC-Tet-B-RRXS$_{20}$ after incubation with PKA, with expected and observed masses of 3288.0 and 3367.8 Da respectively. The mass is 79.8 Da larger than the peptide on its own due to the addition of a phosphoryl group. (Right) Representative mass spectrum for apCC-Tet-A-S$_{17}$ after incubation with PKA, with expected and observed masses of 3224.5 and 3224.5 Da respectively.

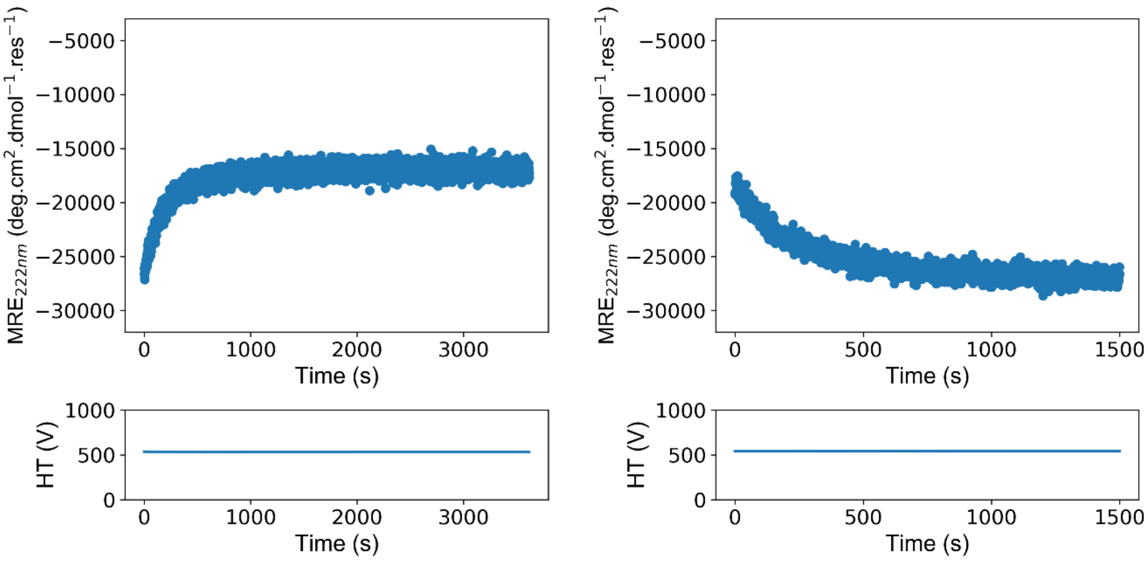

Figure S28. Representative phosphorylation and dephosphorylation reaction for apCC-Tet-A-S_{10} plus apCC-Tet-B-$RRXS_{20}$. Phosphorylation (left) and dephosphorylation (right) at 10 and 9.85 μM individual peptide respectively, with mean residue ellipticity at 222 nm (MRE_{222}) (top), and HT traces (bottom). PKA concentration 5 U/μL, with phosphorylation reaction carried out in phosphorylation buffer. LPP concentration 2 U/μL, with dephosphorylation reaction carried out in phosphorylation buffer plus $MnCl_2$.

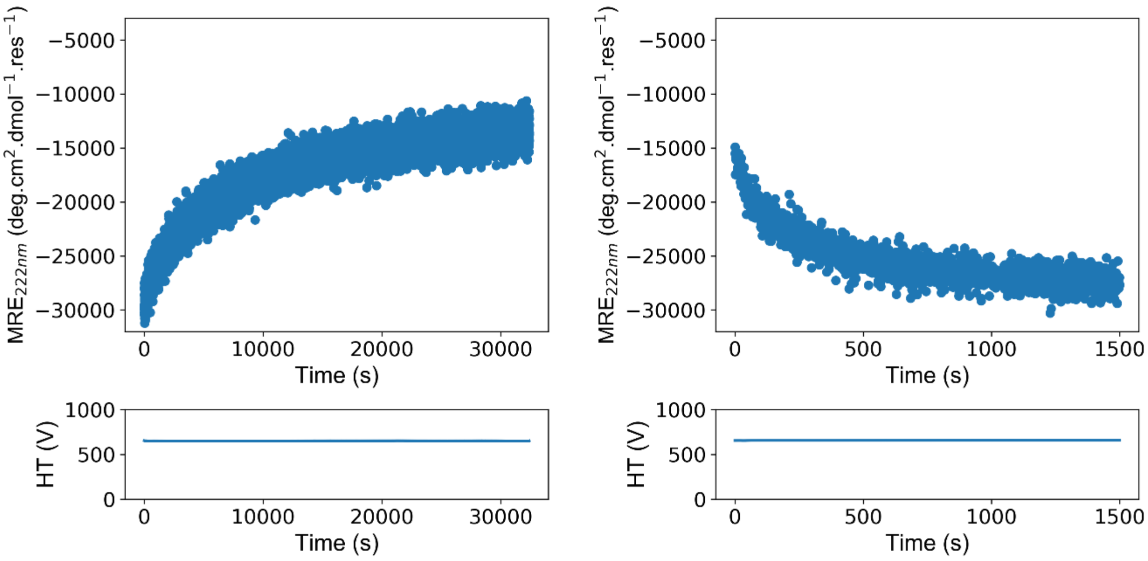

Figure S29. Representative phosphorylation and dephosphorylation reaction for apCC-Tet-A-S_{17} plus apCC-Tet-B-$RRXS_{13}$. Phosphorylation (left) and dephosphorylation (right) at 10 and 9.85 μM individual peptide respectively, with mean residue ellipticity at 222 nm (MRE_{222}) (top), and HT traces (bottom). PKA concentration 5 U/μL, with phosphorylation reaction carried out in phosphorylation buffer. LPP concentration 2 U/μL, with dephosphorylation reaction carried out in phosphorylation buffer plus $MnCl_2$.

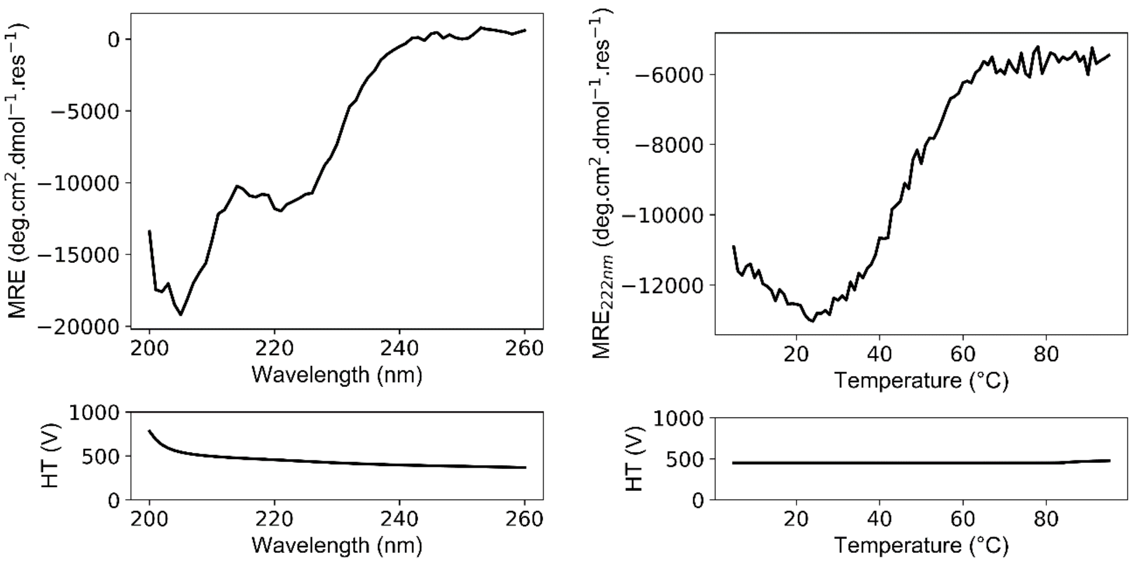

Figure S30. Low concentration CD spectroscopy data for CC-Tet-A-S_{14} and CC-Tet-B-RRXS_{14}. (Left) 5 °C CD spectra data (top) and HT traces (bottom) for the peptides CC-Tet-A-S_{14} plus CC-Tet-B-RRXS_{14}. (Right) Variable temperature (5 – 95 °C) CD measurement (top) and HT traces (bottom) for the peptides CC-Tet-A-S_{14} plus CC-Tet-B-RRXS_{14}, with a midpoint of thermal unfolding (T_M) of 45 °C. Measurements recorded with 1 μM individual peptide in PBS (pH 7.4).

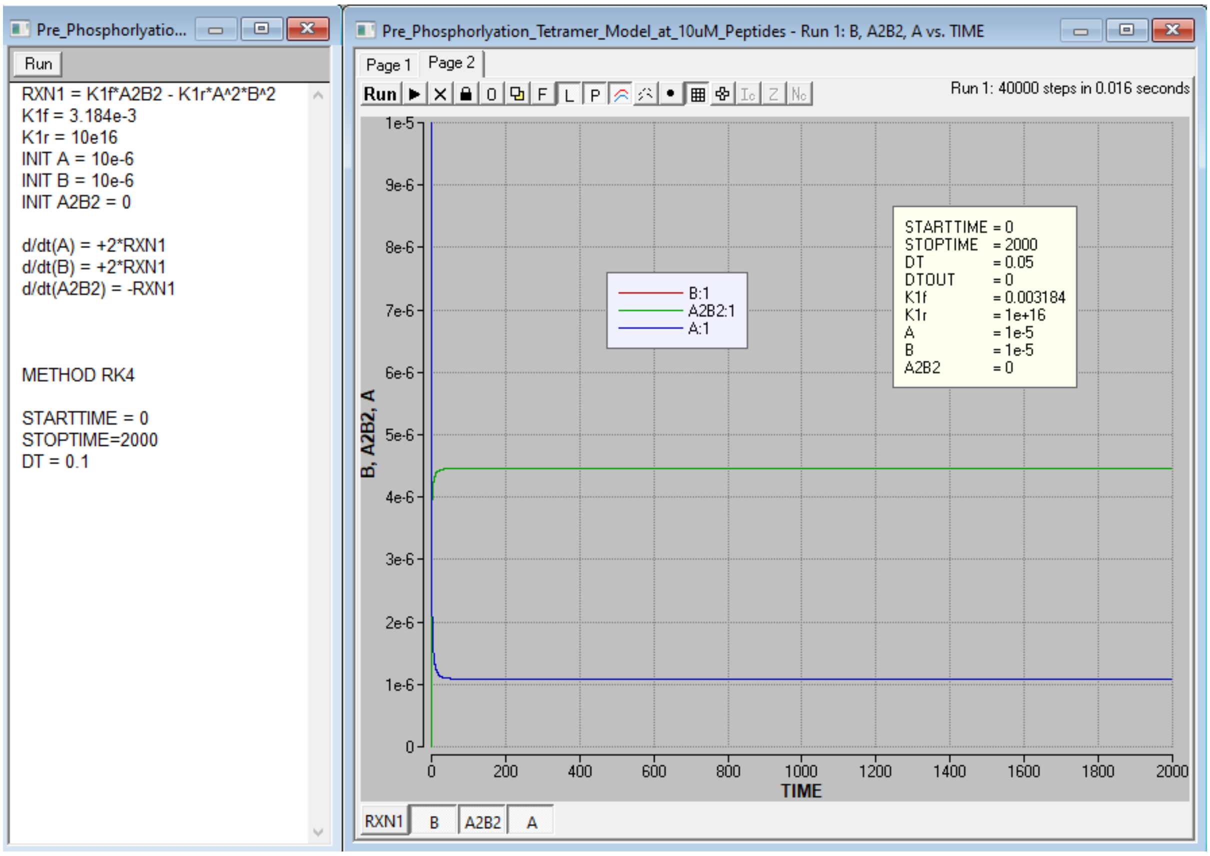

Figure S31. Modelling concentration of tetramer and components at 10 µM individual peptide. Screenshot of Berkeley Madonna model for formation and loss of tetramer displaying model equations (left) and model results and parameters (right). This model provided values of individual unfolded peptides (1.0926 µM), individual folded peptides (8.9074 µM) and folded tetramer (4.4537 µM) in this experiment, which were required as starting points for modelling the phosphorylation reaction.

Supplementary Tables

Table S1: Sequences of antiparallel hetero-tetramer peptide synthesized for this study.[a]

Peptide	Sequence					
**Register**[b]		***gabcdef***	***gabcdef***	***gabcdef***	***gabcdef***	
apCC-Tet	Ac-G	ELEALAQ	ELEALAK	KLKALAW	KLKALAQ	G-NH_2
apCC-Tet-A1	Ac-G	ELEALAQ	ELEALAK	**E**L**E**ALAW	**E**L**E**ALAQ	G-NH_2
apCC-Tet-B1	Ac-G	**K**L**K**ALAQ	**K**L**K**ALAK	KLKALAW	KLKALAQ	G-NH_2
apCC-Tet-A-S_{17}	Ac-G	ELEALAQ	ELEALAK	E**S**EALAW	ELEALAQ	G-NH_2
apCC-Tet-A-S_{10}	Ac-G	ELEALAQ	E**S**EALAK	ELEALAW	ELEALAQ	G-NH_2
apCC-Tet-B-S_{20}	Ac-G	KLKALAQ	KLKALAK	KLKA**S**AW	KLKALAQ	G-NH_2
apCC-Tet-B-$RRXS_{20}$	Ac-G	KLKALAQ	KLKALAK	K**RRAS**AW	KLKALAQ	G-NH_2
apCC-Tet-B-$RRXS_{13}$	Ac-G	KLKALAQ	K**RRAS**AK	KLKALAW	KLKALAQ	G-NH_2
apCC-Tet-A-S_{17} $SeMet_4$	Ac-G	EL**Φ**ALAQ	ELEALAK	E**S**EALAQ	ELEALAQ	G-NH_2
apCC-Tet-B-S_{20} $4CF_2$	Ac-G	**Ω**LKALAQ	KLKALAK	KLKA**S**AQ	KLKALAQ	G-NH_2
apCC-Tet-B-S_{20} $4CF_{25}$	Ac-G	KLKALAQ	KLKALAK	KLKA**S**AQ	KL**Ω**ALAQ	G-NH_2

[a]All peptides were N-terminally acetylated and C-terminally amidated. [b]Heptad registers are given above the sequences. Peptides are in g-register, which refers to the first residue of the peptide excluding the capping Gly. Introduced substitutions are highlighted in bold. Ω = 4-Cyano-L-phenylalanine (4CF) and Φ = Selenomethionine.

Table S2: Sequences of parallel hetero-tetramer peptides synthesized for this study.[a]

Peptide	Sequence						
**Register**		***cdef***	***gabcdef***	***gabcdef***	***gabcdef***	***gab***	
CC-Tet-A	Ac-G	AIEK	ELAAIEK	ELAAIEW	ELAAIEK	ELA	G-NH_2
CC-Tet-A-S_7	Ac-G	AIEK	E**S**AAIEK	ELAAIEW	ELAAIEK	ELA	G-NH_2
CC-Tet-A-S_{14}	Ac-G	AIEK	ELAAIEK	E**S**AAIEW	ELAAIEK	ELA	G-NH_2
CC-Tet-A-S_{21}	Ac-G	AIEK	ELAAIEK	ELAAIEW	E**S**AAIEK	ELA	G-NH_2
CC-Tet-B	Ac-G	AIKQ	KLAAIKQ	KLAAIKW	KLAAIKQ	KLA	G-NH_2
CC-Tet-B-$RRXS_{14}$	Ac-G	AIKQ	KLAAI**RR**	K**S**AAIKW	KLAAIKQ	KLA	G-NH_2

[a]All peptides are in c-register and are N-terminally acetylated and C-terminally amidated. A, acidic; B, basic; Ac, acetyl. Substitutions are highlighted in bold and underlined.

Table S3: Summary of modelling scores for parallel phospho-switching designs.

Design Combination	BUDE FF Score
CC-Tet-A + CC-Tet-A-B-$RRXS_{14}$	-2379.9
CC-Tet-A-S_{14} + CC-Tet-A-B-$RRXS_{14}$	-2343.4
CC-Tet-A-S_7 + CC-Tet-A-B-$RRXS_{14}$	-2341.2
CC-Tet-A-S_{21} + CC-Tet-A-B-$RRXS_{14}$	-2339.6

Modelling conducted in ISAMBARD[1] using parameters optimized from the crystal structure of CC-Tet (PDB entry 3R4A).

Table S4. Biophysical parameters for switchable peptides and complexes.

Peptide(s)	Helicity at 5 °C (%)[a]	T_M (°C)
CC-Tet-A	37[b]	< 20[b]
CC-Tet-B	37[b]	< 20[b]
CC-Tet-A-S_{14}	23	< 20
CC-Tet-A-S_7	29	< 20
CC-Tet-A-S_{21}	25	< 20
CC-Tet-B-RRXS_{14}	26	< 20
CC-Tet-A + B	87[b]	> 95[b]
CC-Tet-A-S_{14} + B-RRXS_{14}	67	62
CC-Tet-A-S_7 + B-RRXS_{14}	70	66
CC-Tet-A-S_{21} + B-RRXS_{14}	81	72
apCC-Tet-A-S_{10}	48	< 20
apCC-Tet-A-S_{17}	42	< 20
apCC-Tet-B-S_{20}	40	< 20
apCC-Tet-B-RRXS_{13}	39	< 20
apCC-Tet-B-RRXS_{20}	38	< 20
apCC-Tet-A-S_{10} + B-S_{20}	93	> 95
apCC-Tet-A-S_{10} + B-RRXS_{20}	80	75
apCC-Tet-A-S_{17} + B-RRXS_{13}	84	72
apCC-Tet-A-S_{17} + B-RRXS_{20}	71	64

[a]*Units, deg.cm^2.dmol^{-1}.res^{-1}.* [b]*Values are taken from*[5]*. Measurements were recorded in PBS (pH 7.4) at 10 μM individual peptide.*

Table S5. Rates of phospho-switching for heterotetramer peptide complexes.

Peptide Combination	Phosphorylation		Dephosphorylation	
	Half Life (s)	Completion (s)	Half Life (s)	Completion (s)
CC-Tet-A-S_{14} + CC-Tet-B-$RRXS_{14}$	290	1500	58	550
CC-Tet-A-S_7 + CC-Tet-B-$RRXS_{14}$	1650	10000	62	600
CC-Tet-A-S_{21} + CC-Tet-B-$RRXS_{14}$	4220	20000	75	650
apCC-Tet-A-S_{17} + apCC-Tet-B-$RRXS_{20}$	110	1200	140	1200
apCC-Tet-A-S_{10} + apCC-Tet-B-$RRXS_{20}$	160	1600	180	1400
apCC-Tet-A-S_{17} + apCC-Tet-B-$RRXS_{13}$	5550	34000	180	1500

These values were calculated based on CD phosphorylation of the peptide combinations using 10 and 9.85 µM of each peptide for phosphorylation and dephosphorylation reactions respectively. Phosphorylation reactions were conducted with 5 U/µl PKA for all combinations except apCC-Tet-A-S_{17} plus apCC-Tet-B-$RRXS_{20}$, for which 1 U/µl PKA was used. All dephosphorylation reactions used 2 U/µl LPP. All reactions were conducted at 37 °C.

Table S6. Variables used in modelling phospho-switching tetramer system.

Variable	Calculated by or taken from
k_{off} = 0.0038 s^{-1}	Values optimized to fit experimental CD phosphorylation data using curve fit feature of Berkeley Madonna, whilst maintaining the K_D as 3.18 x 10^{-19} M^3.
k_{on} = 1.1935 x 10^{16} $M^{-3}s^{-1}$	
Initial [A] = 1.0926 µM	Values calculated from experimentally determined K_D and system model displayed in Figure S31.
Initial [B] = 1.0926 µM	
Initial [A_2B_2] = 4.4537 µM	
Initial [Bp] = 0 µM	
Initial [K] = 105 nM	Concentration of PKA used in CD phosphorylation experiments.
k_{phos} = 1064000 $M^{-1}s^{-1}$	Used value of k_{cat}/K_M from published values of PKA phosphorylation of Kemptide[6], given the sequence similarity to CC-Tet-B-$RRXS_{14}$.

Supplementary References

1. Wood, C. W.; Heal, J. W.; Thomson, A. R.; Bartlett, G. J.; Ibarra, A. Á.; Brady, R. L.; Sessions, R. B.; Woolfson, D. N.; Valencia, A., ISAMBARD: an open-source computational environment for biomolecular analysis, modelling and design. *Bioinformatics* **2017,** *33* (19), 3043-3050.
2. Zaccai, N. R.; Chi, B.; Thomson, A. R.; Boyle, A. L.; Bartlett, G. J.; Bruning, M.; Linden, N.; Sessions, R. B.; Booth, P. J.; Brady, R. L.; Woolfson, D. N., A de novo peptide hexamer with a mutable channel. *Nat Chem Biol* **2011,** *7* (12), 935-41.
3. *The PyMOL Molecular Graphics System*, 2.5 Schrödinger, LLC: 2021.
4. Rhys, G. G.; Wood, C. W.; Beesley, J. L.; Zaccai, N. R.; Burton, A. J.; Brady, R. L.; Thomson, A. R.; Woolfson, D. N., Navigating the Structural Landscape of De Novo α-Helical Bundles. *J. Am. Chem. Soc.* **2019,** *141* (22), 8787-8797.
5. Edgell, C. L.; Smith, A. J.; Beesley, J. L.; Savery, N. J.; Woolfson, D. N., De Novo Designed Protein-Interaction Modules for In-Cell Applications. *ACS Synth. Biol.* **2020,** *9* (2), 427-436.
6. Moore, M. J.; Adams, J. A.; Taylor, S. S., Structural basis for peptide binding in protein kinase A - Role of glutamic acid 203 and tyrosine 204 in the peptide-positioning loop. *J. Biol. Chem.* **2003,** *278* (12), 10613-10618.
